# Increased Expression of the Dyslexia Candidate Gene DCDC2 Affects Length and Signaling of Primary Cilia in Neurons

**DOI:** 10.1371/journal.pone.0020580

**Published:** 2011-06-16

**Authors:** Satu Massinen, Marie-Estelle Hokkanen, Hans Matsson, Kristiina Tammimies, Isabel Tapia-Páez, Vanina Dahlström-Heuser, Juha Kuja-Panula, Jan Burghoorn, Kristian E. Jeppsson, Peter Swoboda, Myriam Peyrard-Janvid, Rune Toftgård, Eero Castrén, Juha Kere

**Affiliations:** 1 Research Program's Unit, Molecular Medicine and Department of Medical Genetics, University of Helsinki, Helsinki, Finland; 2 Neuroscience Center, University of Helsinki, Helsinki, Finland; 3 Department of Biosciences and Nutrition, Karolinska Institutet, Huddinge, Sweden; 4 School of Life Sciences, Södertörn University College, Huddinge, Sweden; 5 Folkhälsan Institute of Genetics, Helsinki, Finland; Instituto de Ciencia de Materiales de Madrid - Instituto de Biomedicina de Valencia, Spain

## Abstract

*DCDC2* is one of the candidate susceptibility genes for dyslexia. It belongs to the superfamily of doublecortin domain containing proteins that bind to microtubules, and it has been shown to be involved in neuronal migration. We show that the Dcdc2 protein localizes to the primary cilium in primary rat hippocampal neurons and that it can be found within close proximity to the ciliary kinesin-2 subunit Kif3a. Overexpression of DCDC2 increases ciliary length and activates Shh signaling, whereas downregulation of Dcdc2 expression enhances Wnt signaling, consistent with a functional role in ciliary signaling. Moreover, DCDC2 overexpression in *C. elegans* causes an abnormal neuronal phenotype that can only be seen in ciliated neurons. Together our results suggest a potential role for DCDC2 in the structure and function of primary cilia.

## Introduction

The neurobiology of dyslexia, the most common learning disability, remains poorly understood but accumulated evidence suggests that dyslexia may be associated with impaired neuronal migration or axonal guidance. Genetic studies have identified several variants within or near the Doublecortin domain containing 2 (*DCDC2*) gene that are associated to dyslexia [1]. Together with two other dyslexia susceptibility candidate genes (dyslexia susceptibility 1 candidate 1, *DYX1C1*, and *KIAA0319*), Dcdc2 has been shown to be involved in neuronal migration in the developing cortex in rats [2]–[5]. In addition, *ROBO1*, a human ortholog of the axon guidance receptor roundabout, has been implicated in dyslexia and has also been shown to participate in the migration of cortical interneurons [6], [7]. DCDC2 belongs to the superfamily of doublecortin domain containing proteins that regulate cytoskeletal dynamics by binding to and stabilizing microtubules (Fig. S1) [8], [9]. Nevertheless the cellular function of DCDC2 remains unclear.

The primary cilium is an organelle found on most cells in different tissues throughout the mammalian body including the central nervous system [10], [11]. The cilium consists of an array of microtubules, called the axoneme, constructed on a basal body that is based on one of the centrioles. The axoneme is ensheathed by the ciliary membrane, an extension of the plasma membrane, which is characterized by specific membrane receptors. Ciliary proteins are synthesized in the cytoplasm and endoplasmic reticulum and transported within the cilium by a machinery called intraflagellary transport (IFT) that includes motor proteins, kinesins and dyneins. Several important developmental signaling pathways have been associated with cilia including Sonic hedgehog, Wnt and Platelet derived growth factor [10]. The function of the primary cilium in neurons is not clear but it has been shown to have a role in cortical morphogenesis [12] and adult neurogenesis [13]. Many human genetic diseases have recently been linked to malfunctions of the primary cilium [14].

In this study we have investigated the cellular function of the dyslexia candidate protein DCDC2 in neurons. We show that DCDC2 localizes to the primary cilium when overexpressed and that DCDC2 affects ciliary signaling. Moreover overexpression of DCDC2 influences the morphology of ciliated cells in vivo in *C. elegans* and mammalian cells *in vitro*.

## Results

### DCDC2 localizes to the primary cilium

**Figure 1:**
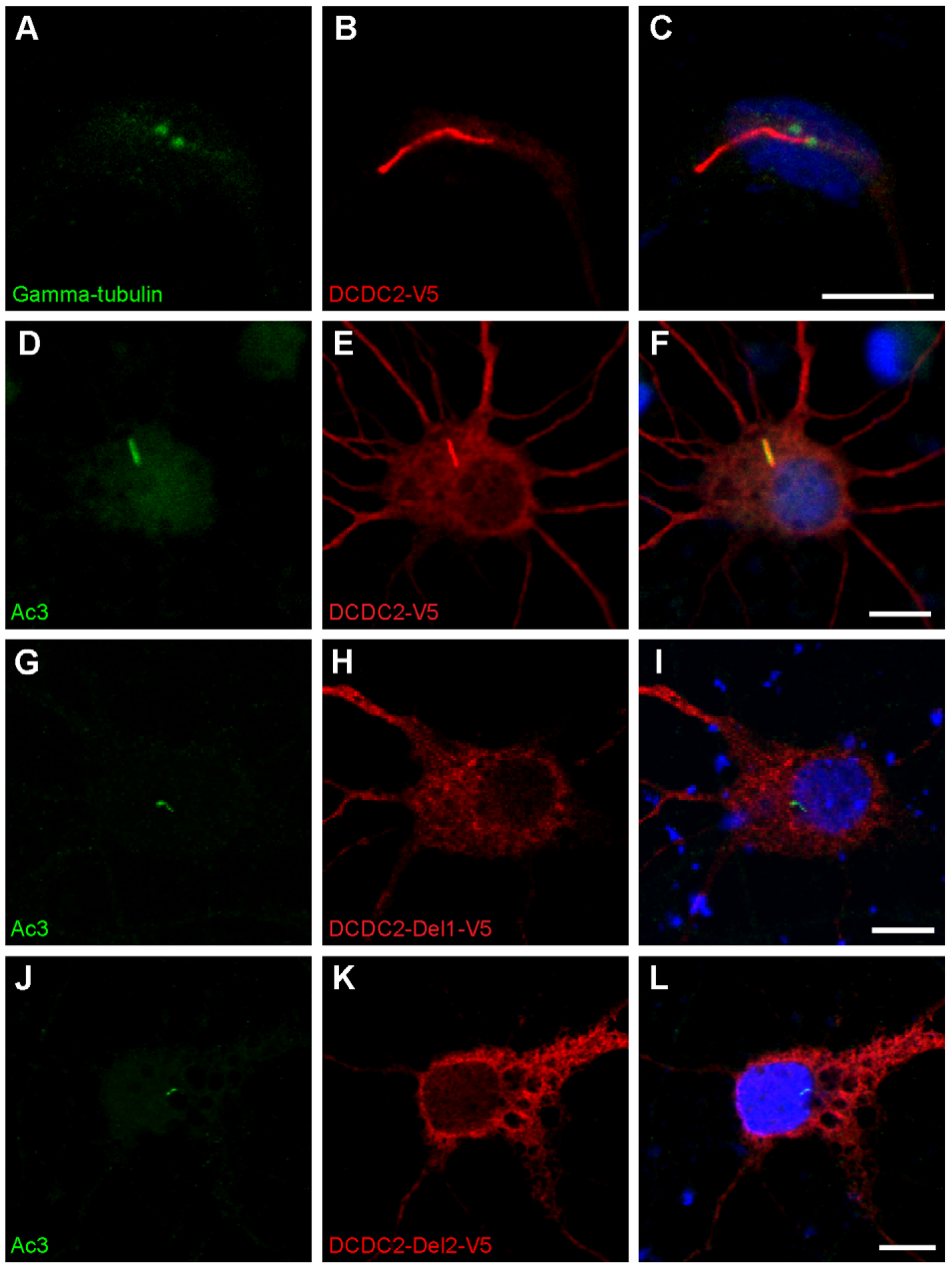
Full-length V5-tagged DCDC2 localizes to the primary cilium in neurons. Confocal images of rat primary hippocampal neurons transfected with DCDC2-V5 and labeled with a centriolar marker gamma-tubulin (A) or the neuronal ciliary marker Ac3 (D) and the V5 epitope (B and E). Nuclei were stained with DAPI (blue). The merged image shows colocalization of Ac3 and DCDC2-V5 in the primary cilium (F). Neurons transfected with deletion constructs of DCDC2 lacking either of the two doublecortin domains do not show ciliary localization of the protein (G–L). Scale bars indicate 10 µm.

To study the subcellular localization of DCDC2 we transfected primary rat hippocampal neurons with an expression construct expressing human DCDC2 tagged with either GFP or V5. Immunocytochemical stainings against the tags revealed that DCDC2 was localized to the primary cilium, neurites and the cytoplasm. The ciliary localization was verified by double staining for markers of neuronal cilia (adenylate cyclase 3 [Ac3]) and the centrioles at the base of the cilium (gamma-tubulin) (Fig. 1A–F). The neuronal phenotype of the majority of the transfected cells was confirmed by immunostaining for beta-tubulin III (clone Tuj1) and neuronal nuclear antigen (NeuN) (Fig. S2). The ciliary localization of DCDC2 was also observed in mouse NIH/3T3 fibroblast cells, which are ciliated when the cells are not dividing. This localization was confirmed by double staining of the V5-tag together with the ciliary marker acetylated tubulin and the centrosomal marker DNAL4. In nonciliated cells (such as dividing NIH/3T3 cells and COS-7 cells), DCDC2 accumulated in the cytoplasm on microtubule networks near the centrosome (Fig. S3).

**Figure 2:**
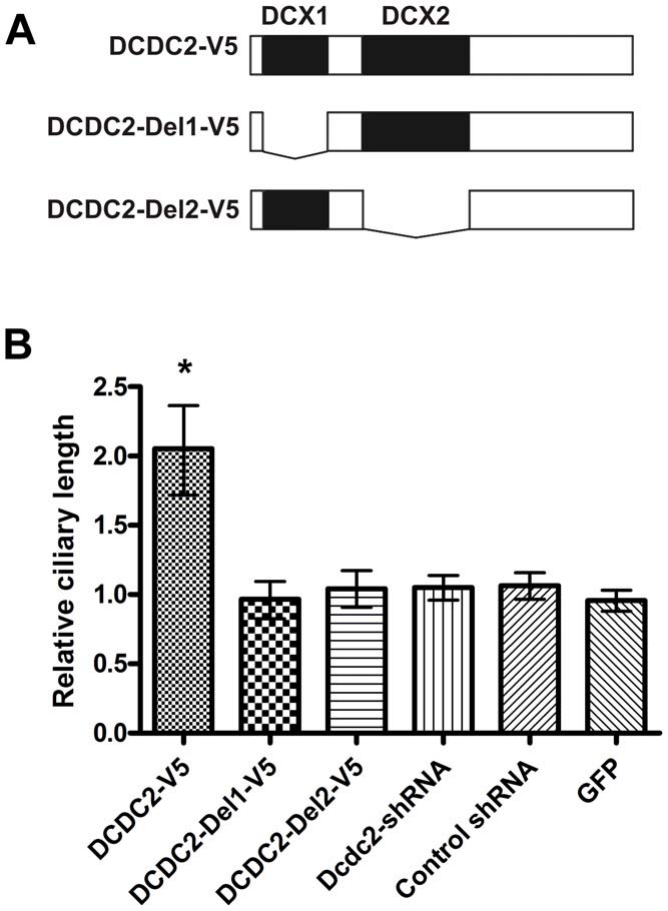
Overexpression of DCDC2 enhances primary cilia length. Schematic illustration of the DCDC2 deletion constructs (A). Doublecortin domains (DCX1 and DCX2) are shown as black boxes. Overexpression of DCDC2 increases ciliary length in hippocampal neurons (B). Ciliary length of transfected cells (t-test *p = 2,2×10^−9^; +/−95% confidence intervals) relative to the cilia in untransfected cells.

We next investigated the role of the doublecortin domains in the ciliary localization using deletion constructs of DCDC2 that lacked either of the doublecortin domains (Fig. 2A). We were not able to detect a ciliary localization for the deletion constructs in hippocampal neurons or NIH/3T3 cells, which suggests that both doublecortin domains may be needed for the ciliary localization. (Fig. 1 G–L). However, both of the deletion constructs were able to bind to microtubules in pelleting assays (Fig. S1). The deletion constructs did not appear to have any dominant negative effect, since the full-length construct still localized to the cilium when coexpressed with the deletion constructs (Fig. S4).

### Overexpression of DCDC2 increases ciliary length

The length of the cilium is controlled by microtubule dynamics [15]. We hypothesized that if DCDC2 stabilizes microtubules in the cilium, the overexpression of DCDC2 might impact on ciliary length by making microtubule depolymerisation less favorable. Moreover another doublecortin family protein, RP1(9), has been shown to localize to the photoreceptor connecting cilium in mouse and regulate the length of the microtubule scaffold in this modified cilium *in vitro* and *in vivo* [16]

Indeed, the average length of the cilium was approximately twice as long in hippocampal neurons overexpressing DCDC2 as in untransfected cells in the same cultures (Fig. 2B). The increase in ciliary length could also be seen in NIH/3T3 cells overexpressing DCDC2 (Fig. S5). Both the cytoskeleton and the membrane were extended when DCDC2 was overexpressed, as revealed by a staining against acetylated tubulin in the axoneme in NIH/3T3 cells and Ac3 in neuronal membranes. In contrast, Dcdc2 knockdown by shRNA or transfection with DCDC2 deletion constructs had no effect on ciliary length (Fig. 2B).

### DCDC2 associates with Kif3a at the primary cilium

Intraflagellar transport (IFT), the molecular transport system within the cilium, is essential in the formation, maintenance, length control and signaling functions of the cilium [17]. During IFT, cargo is transported bidirectionally along microtubules by molecular motors, kinesin (anterograde transport) and dynein (retrograde transport). The ciliary motor proteins function as complexes with associated proteins.

**Figure 3:**
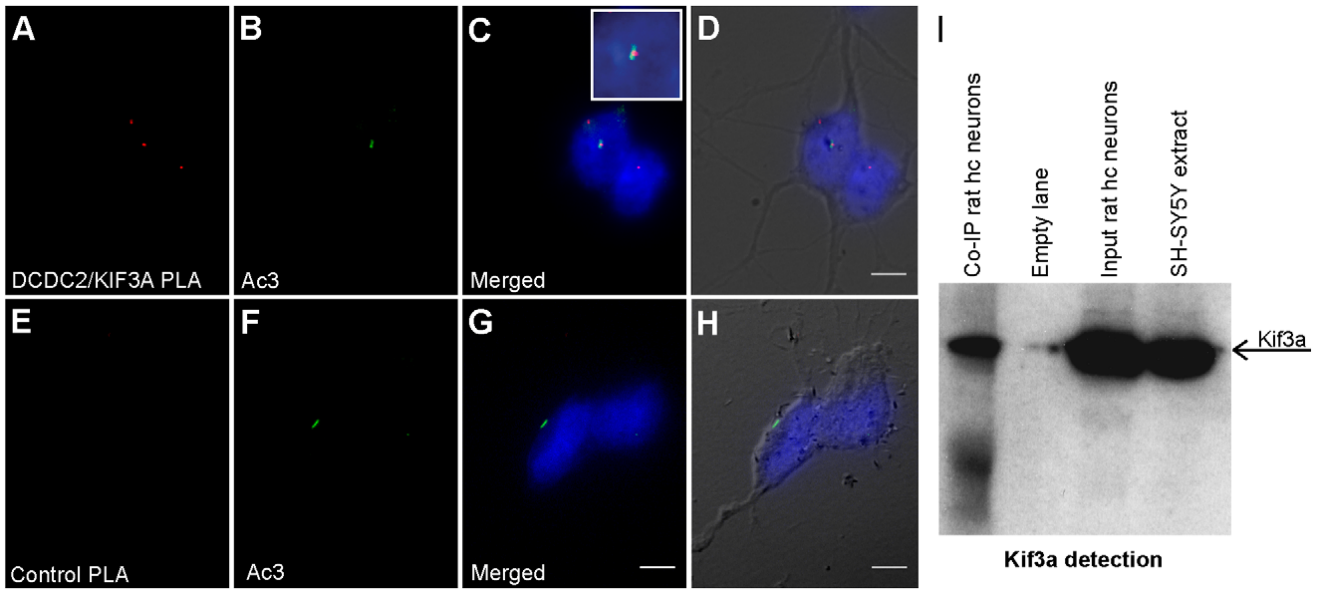
Dcdc2 associates with Kif3a in the primary cilium and the cytosol. Dcdc2/Kif3a complexes are detected in cultured rat hippocampal neurons with *in situ* proximity ligation assay (*in situ* PLA) in which the close physical association of two proteins are visualized as a red fluorescent signal (A). An antibody against adenylyl cyclase (Ac3) is used to stain cilia (B, F). The Dcdc2/Kif3a protein complex co-localizes with Ac3. Merged images of the *in situ* PLA products and Ac3 staining are shown in (C). Digital magnification of the area of co-localized signals is shown in the white box. The specificity of the assay was confirmed by a control assay (E–H) without the primary antibody against DCDC2 and no background signal from PLA products can be detected. The merged images (C,G) are shown overlapping with bright field images from the same position (D,H). Nuclei were stained with Hoechst 33432 (blue). Scale bars indicate 10 µm. Part I shows co-immunoprecipitation experiment demonstrating the association between Kif3A and Dcdc2 *in vitro*. The first lane was loaded with rat hippocampal (hc) neurons immunoprecipitated with a Dcdc2 antibody (see Materials and Methods). Total protein extracts from rat hc neurons (input) and neuroblastoma cell line SH-SY5Y are shown in lane number three and four. The filter was probed with an antibody against KIF3A (arrow).

One of the most important ciliary proteins is Kif3a, a component of kinesin-2, which is essential for ciliary formation and function. To test whether DCDC2 might be linked to the function of molecular motors in the cilium we determined the physical association between endogenous Kif3a and Dcdc2 in rat hippocampal neurons by *in situ* proximity ligation assay (*in situ* PLA). This PLA method can identify protein complexes at native levels with high sensitivity [18]. In the *in situ* PLA primary antibodies raised in different species are used against the proteins of interest. Species-specific oligonucleotide conjugated secondary antibodies (PLA probes) bind to the primary antibodies. Subsequently, an oligonucleotide is annealed to complementary DNA of PLA probes in close proximity that after ligation allow rolling circle amplification. The signal from the close physical association between the two proteins of interest is produced by a fluorescent probe complimentary to a sequence in the amplified product and visualized here as red dots. As shown in figure 3, clear positive PLA signal was detected over the primary cilium, indicating that the Dcdc2 and Kif3a proteins can be found within close proximity in the cilium. A positive signal was also observed in the cytoplasm, which is consistent with the expression of both Dcdc2 and Kif3a in the cytoplasm as well as in the cilia and suggests that the colocalization of these two proteins is not confined to the cilia.

To confirm the association between Dcdc2 and Kif3a we also performed a co-immunopreciptation assay (Fig. 3I). Lysates from rat primary hippocampal neurons were pulled down with an antibody against DCDC2. Probing with Kif3a antibody revealed that Kif3a could be precipitated together with Dcdc2. The immunoprecipitate was also probed with the DCDC2 antibody to confirm the antibody's specificity for Dcdc2 (Fig. S6). Moreover the antibody was able to detect the overexpressed protein in western blots and in primary hippocampal cells (Fig. S6).

### DCDC2 affects ciliary signaling in primary neuronal cultures

The primary cilium regulates several important signaling pathways, including the Sonic hedgehog (Shh) and Wnt pathways which have been shown to play an important role in early patterning during development both in the nervous system and elsewhere. In the adult CNS both Shh and Wnt pathways seem to be important for neurogenesis [19], [20] Recently they have also been shown to be important for the postmitotic development of neurons affecting axonal guidance [21], [22]. Wnt signaling has also been found to be involved in synapse formation [21]. The key proteins in both Shh and Wnt signaling are also expressed in the adult CNS in other locations than the neurogenic niches [23], [24] but the function of these pathways in other aspects than neurogenesis of the adult CNS remains poorly understood.

Kif3a has been shown to be a key molecule in both Shh and Wnt pathways. Patched (Ptc), the receptor for Shh, is localized in the cilium and it prevents Smoothened (Smo) from accumulating in the cilium. When Ptc binds Shh, it moves out of the cilium, which allows the Kif3a mediated translocation and accumulation of Smo into the cilia and transduction of the Shh signal through Gli transcriptional regulators [25]. In the Wnt signaling pathway Kif3a can function through a protein network including Dishevelled (Dvl) in regulating the phosphorylation and stabilization of beta-catenin that interacts with TCF/Lef transcription factors to activate transcription [26].

**Figure 4:**
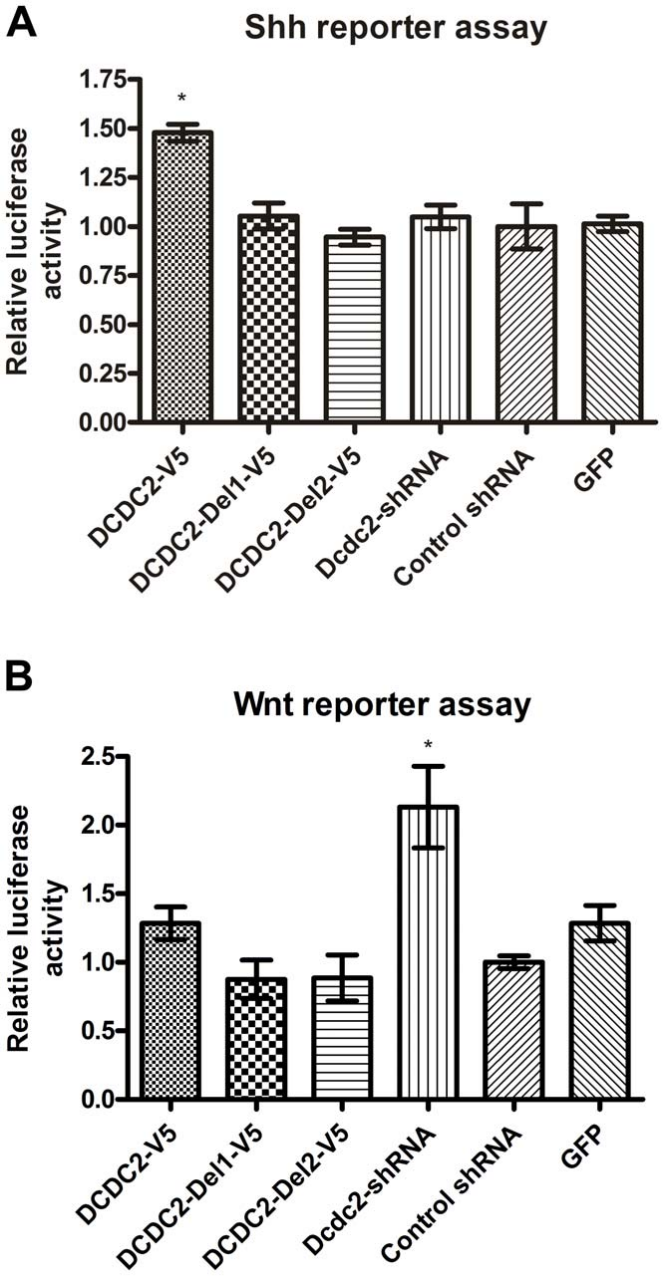
Dcdc2 affects ciliary signaling. Overexpression of DCDC2 activates Shh signaling (A). DCDC2 overexpressing neurons had more Shh pathway activation compared to neurons transfected with DCDC2 deletion constructs, Dcdc2-shRNA or control vectors (ANOVA followed by T-test, * p = 7,3×10^−5^). Dcdc2 knockdown by shRNA activates canonical Wnt signaling (B) as measured by a reporter luciferase assay (ANOVA, followed by T-test * p = 3,7×10^−5^). The error bars indicate SEM.

To study the role of DCDC2 in Shh signaling, we cotransfected rat primary cortical neurons with luciferase reporter vectors for the Shh pathway together with the DCDC2 full-length or deletion constructs or Dcdc2-shRNA. Transfection with the full-length DCDC2 significantly increased Shh signaling but the deletion constructs or the knockdown of DCDC2 had no effect (Fig. 4A). Our results suggest that overexpression of DCDC2 may enhance the Kif3a mediated translocation of Smo to the cilium, resulting in overactivation of the Shh pathway.

The primary cilium is not required for Wnt signaling, but in ciliated cells, the role of the cilium is to restrain canonical Wnt signaling. An increase in canonical Wnt signaling has been seen when the function of the primary cilium has been disrupted by loss of Kif3a or two other ciliogenic genes [26]. To study the effect of DCDC2 on canonical Wnt signaling we used a luciferase reporter assay responsive to TCF/Lef activation. Transfection with full-length DCDC2 or the deletion constructs had no effect on canonical Wnt signaling. Instead a significant activation of canonical Wnt signaling could be seen after knockdown of Dcdc2 with the Dcdc2-shRNA, which could be prevented by simultaneously expressing DCDC2 (Fig. 4B and Fig. S7).

### Microarray analysis of DCDC2 overexpression

We also investigated possible alterations at the transcriptome level resulting from over-expressing DCDC2 in hippocampal neurons and control neurons using the Affymetrix Rat Gene ST 1.0 array. A total of 54 genes were up- or downregulated (Table 1). The gene ontology (GO) analysis of the differentially expressed genes revealed significant enrichment for cellular component microtubule cytoskeleton (adj. p-value 2.49×10^−5^) and biological process cell cycle related terms (Fig. S8). In pathway analysis using Wikipathways, we found that cell cycle (adj. p-value 2.0×10^−3^) and Hedgehog signaling pathway (adj. p-value 2.0×10^−3^) were significantly enriched supporting our hypothesis of the function of DCDC2. Interestingly, the most up-regulated gene was platelet derived growth factor receptor, alpha polypeptide (*Pdgfrα*) (Table 1), the signaling of which is also coordinated by the primary cilium [27]. Key microarray results were further confirmed by quantitative real-time PCR (qRT-PCR). For the verification, we selected five genes: *Pdgfra* which was the most upregulated gene, *Cdc2* which is involved in the same pathway as *Pdgfra*, two kinesin family genes *Kif4* and *Kif2c*, and *Hhip* that is involved in Sonic Hedgehog signaling. Changes in gene expression measured by qRT-PCR were fully consistent with the data obtained by microarray analysis (Fig. S9).

**Table 1:** A list of differentially expressed genes with B-values more than 1.

*Gene Symbol*	*Gene name*	*Entrez ID*	*FC*	*p-value*	*adjusted p-value*	*B*
Pdgfra	platelet derived growth factor receptor, alpha polypeptide	25267	2,27	2,07E-06	0,0282	4,76
Calcrl	calcitonin receptor-like	25029	1,51	5,59E-06	0,0282	4,06
Hist1h1a	histone cluster 1, H1a	291145	1,78	5,71E-06	0,0282	4,04
Arhgap11a	Rho GTPase activating protein 11A	296060	1,74	8,74E-06	0,0282	3,72
LOC680498	similar to CG31613-PA	679950	1,64	8,79E-06	0,0282	3,72
Ttk	Ttk protein kinase	315852	1,60	1,08E-05	0,0282	3,56
Pttg1	pituitary tumor-transforming 1	64193	1,64	1,16E-05	0,0282	3,50
Rad51	RAD51 homolog (RecA homolog, E. coli) (S. cerevisiae)	499870	1,57	1,18E-05	0,0282	3,50
Top2a	topoisomerase (DNA) II alpha	360243	1,90	2,00E-05	0,0372	3,08
Btg1	B-cell translocation gene 1, anti-proliferative	29618	1,47	2,23E-05	0,0372	2,99
Dll3	delta-like 3 (Drosophila)	114125	1,53	2,31E-05	0,0372	2,96
Ankrd15	ankyrin repeat domain 15	309429	1,71	2,33E-05	0,0372	2,96
Hist1h4m	histone cluster 1, H4m	291152	1,67	2,86E-05	0,0404	2,79
Mzf1	myeloid zinc finger 1	361508	−1,44	2,95E-05	0,0404	2,76
Pcdhb15	protocadherin 15	690865	1,47	3,28E-05	0,0419	2,68
Usp1	ubiquitin specific peptidase 1	313387	1,41	4,47E-05	0,0513	2,42
Cdc2	cell division cycle 2, G1 to S and G2 to M	54237	1,76	4,55E-05	0,0513	2,41
Mcm3	minichromosome maintenance complex component 3	316273	1,60	5,34E-05	0,0548	2,27
Hist1h2ao	histone cluster 1, H2ao	364723	1,45	5,43E-05	0,0548	2,26
Cenpf	centromere protein F	257649	1,48	6,73E-05	0,0585	2,07
Epn1	Epsin 1	117277	−1,38	7,25E-05	0,0585	2,01
KIFC2	kinesin family member C2	300053	−1,37	7,27E-05	0,0585	2,01
Myo6	myosin VI	315840	1,35	7,63E-05	0,0585	1,97
Lgr5	leucine rich repeat containing G protein coupled receptor 5	299802	1,56	7,79E-05	0,0585	1,95
Rpl23a	ribosomal protein L23a	360572	−1,68	8,32E-05	0,0585	1,89
Gpsm2	G-protein signaling modulator 2 (AGS3-like, C. elegans)	362021	1,45	8,35E-05	0,0585	1,89
Mki67	antigen identified by monoclonal antibody Ki-67	291234	1,77	8,70E-05	0,0585	1,85
Dlgap5	discs, large (Drosophila) homolog-associated protein 5	289997	1,49	8,79E-05	0,0585	1,84
Fam70b	family with sequence similarity 70, member B	290877	1,76	8,85E-05	0,0585	1,84
Tpx2	TPX2, microtubule-associated, homolog (Xenopus laevis)	311546	1,36	9,66E-05	0,0597	1,76
Obox6	oocyte specific homeobox 6	292629	1,42	9,91E-05	0,0597	1,74
LOC502125	similar to Histone H2A.l (H2A/l)	502125	1,73	9,97E-05	0,0597	1,74
Lhfpl3	lipoma HMGIC fusion partner-like 3	499977	1,44	0,000106	0,0618	1,68
Nxt1	NTF2-like export factor 1	296219	1,37	0,000113	0,0630	1,63
Prc1	protein regulator of cytokinesis 1	308761	1,47	0,000115	0,0630	1,61
Dynlrb1	dynein light chain roadblock-type 1	170714	−1,41	0,000127	0,0662	1,52
RGD1565149	similar to chromosome 16 open reading frame 7	307923	−1,37	0,000128	0,0662	1,52
Ephx1	epoxide hydrolase 1, microsomal	25315	−1,59	0,000131	0,0662	1,50
Sst	Somatostatin	24797	−1,40	0,000136	0,0668	1,47
Kif4	kinesin family member 4	84393	1,59	0,000153	0,0710	1,36
Cmtm6	CKLF-like MARVEL transmembrane domain containing 6	316035	1,32	0,000155	0,0710	1,35
RGD1305939	hypothetical LOC300074	300074	−1,81	0,000156	0,0710	1,35
Fcho1	FCH domain only 1	290639	−1,48	0,000161	0,0718	1,32
Hist2h2bb	histone cluster 2, H2bb	295278	1,52	0,000175	0,0762	1,24
Mrpl18	ribosomal protein L18	81766	1,38	0,00018	0,0768	1,21
Ranbp10	RAN binding protein 10	361396	−1,30	0,000192	0,0799	1,16
Hhip	Hedgehog-interacting protein	291936	1,34	0,000208	0,0801	1,09
Twf1	twinfilin, actin-binding protein, homolog 1 (Drosophila)	315265	1,32	0,000211	0,0801	1,07
Palm	Paralemmin	170673	−1,34	0,000213	0,0801	1,07
Leng8	Leukocyte receptor cluster (LRC) member 8	361506	−1,36	0,000216	0,0801	1,05
Cdc20	cell division cycle 20 homolog (S. cerevisiae)	64515	1,50	0,000219	0,0801	1,04
RGD1310784	similar to RIKEN cDNA 2810433K01	291441	1,31	0,000222	0,0801	1,03
Zfp187	zinc finger protein 187	266792	−1,38	0,000223	0,0801	1,02
Kif2c	kinesin family member 2C	171529	1,39	0,000229	0,0801	1,00

### Overexpression of DCDC2 or ZYG-8 leads to aberrant neuronal morphology in *C.elegans*

**Figure 5:**
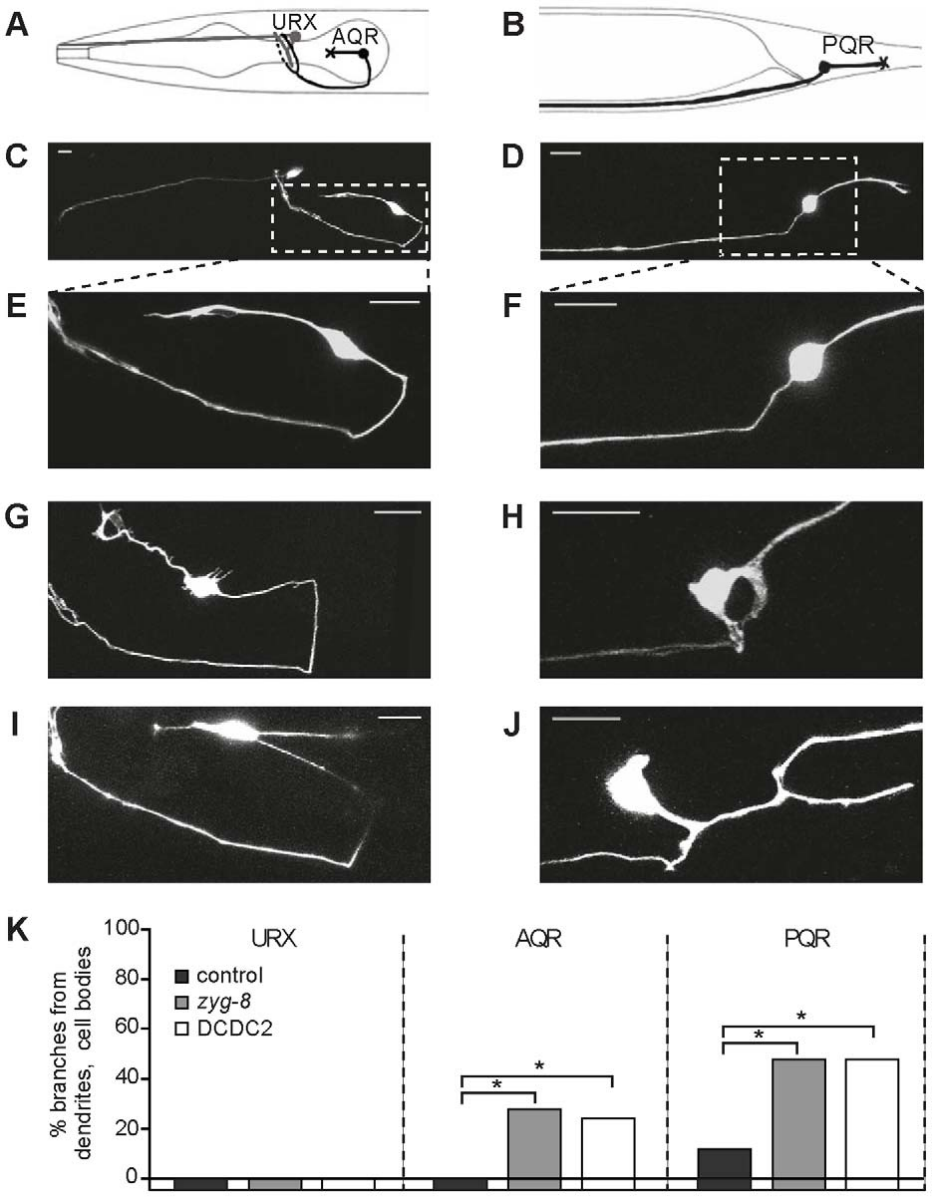
Expression of ZYG-8 or DCDC2 leads to ectopic branching in ciliated neurons of *C. elegans*. The effect of overexpression was observed in three single neurons; the non-ciliated sensory neuron URX (A) in the head and the ciliated sensory neurons AQR (A) in the head and PQR (B) in the tail (pictures adapted from www.wormatlas.org). The X on AQR and PQR represents the position of the cilium in (A) and (B). Neurons transgenic for empty control vector and gcy-32::gfp for visualization show normal morphology; URX is shown in (C), AQR in (C) and (E) and PQR in (D) and (F). Ciliated neurons show an aberrant morphology of dendrites and cell somas when overexpressing either *zyg-8*, AQR in (G) and PQR in (H), or DCDC2 transgene, AQR in (I) and PQR in (J). The quantification (K) shows a significant difference (Fisher's Exact Test, * p<0.03) between genotypes in ciliated (AQR and PQR) but not in non-ciliated (URX) neurons.

To assay the function of DCDC2 in ciliated neurons *in vivo*, we created transgenic *C. elegans* strains overexpressing the human DCDC2 protein. We also created a strain overexpressing the *C. elegans* protein ZYG-8 that, like DCDC2, contains two doublecortin domains and is the only endogenous member of the doublecortin family of proteins in *C.elegans*. Silencing of ZYG-8 during anaphase in the one-cell stage *C. elegans* embryo results in incorrect cell division due to lack of stabilization of microtubules by ZYG-8 [28]. DCDC2 and ZYG-8 were overexpressed in two neurons that are ciliated in the wild-type *C.elegans*, AQR and PQR, and one nonciliated neuron, URX, for comparison (Fig. 5 A, B). Overexpression of human DCDC2 in the ciliated neurons induced ectopic branching at the cell soma and dendrites. On the contrary, DCDC2 overexpression caused no visible phenotype in the nonciliated cells (Fig. 5 C–K). A very similar phenotype was observed when ZYG-8 was overexpressed in ciliated cells whereas nonciliated cells expressing ZYG-8 showed normal phenotype (Fig. 5 C–K). The observation that ciliated neurons were affected but nonciliated neurons were not suggested that the overexpression of ZYG-8 or DCDC2 may disrupt ciliary signaling pathways, leading to an aberrant neuronal morphology. It is interesting to note that in mouse, two doublecortin family proteins Dcx and Dclk have been shown to be involved in the control of dendritic morphology and axon elongation [29].

We also studied the ciliary morphology of the DCDC2 and ZYG-8 overexpressing neurons in *C. elegans* by coexpressing a red fluorescent ciliary marker in AQR and PQR neurons together with either DCDC2 or with ZYG-8. Control neurons not transgenic for DCDC2 or ZYG-8 showed normal cilia at the tip of dendrites. In contrast, cilia could not be seen in neurons overexpressing either of the proteins.

Furthermore, we tested the hypothesis that DCDC2 overexpression would result in a change in neuronal morphology in mammalian cells that would be similar to the change after DCDC2 overexpression in *C.elegans*. We therefore analyzed the total neurite outgrowth and neuronal branching in primary rat hippocampal neurons overexpressing DCDC2-V5 and GFP or GFP only (Fig. S10). In agreement with the findings in *C.elegans* we could see an aberrant morphology of neurite outgrowth as an increase in the branching in rat hippocampal cells. The total length of neurites was not significantly changed.

## Discussion

We show here that the dyslexia candidate protein DCDC2 localizes to the primary cilium in primary neurons. Interestingly, Dcdc2 has been detected in a proteomic analysis of isolated rat olfactory cilia [30]. Moreover, we show that overexpression of DCDC2 affects ciliary length and Shh signaling while knock-down of the endogenous protein in neurons increases Wnt signaling. Furthermore DCDC2 induces aberrant neurite outgrowth specifically in ciliated neurons in *C. elegans in vivo* as well as in rat primary hippocampal neurons *in vitro*.

Our results indicate that Dcdc2 associates with Kif3a in neurons both in the cilium and in the cytoplasm. Kif3a is an essential subunit for transport along microtubules both in the cilium and the cytoplasm in neurons. In the cytoplasm of neurons, the absence of either of the two doublecortin family proteins DCX or DCLK has been shown to disrupt axonal transport [29], a process that also requires the activity of dyneins and kinesins. Moreover, DCX is found with dynein in a protein complex that mediates the coupling of the centrosome to the nucleus in neuronal migration [29].

Cilia play a central role in Shh and Wnt signaling pathways, two pathways important in neuronal development. We show that overexpression of DCDC2 activates Shh signaling while knock-down of the endogenous protein activates canonical Wnt signaling. Therefore, while Dcdc2 knockdown produced no apparent morphological changes in the cilia, the fact that canonical Wnt signaling was enhanced suggested that the function of the cilium was nevertheless compromised. These signaling effects could be Kif3a dependent since both Wnt and Shh signaling pathways have been linked to Kif3a [26], [31]. When overexpressing DCDC2 we could also detect significant changes in the expression levels of highly relevant genes for the cilia signaling and intraflagellary transport, such as *Pdgfra* and kinesin family genes *Kif4* and *Kif2c*.

Overexpression of human DCDC2 or ZYG-8, a *C. elegans* gene also containing two doublecortin domains, induces ectopic dendritic branching in *C. elegans in vivo*. This effect was specific to neurons that are ciliated in the wild-type strain and could not be observed in a neuron that does not have a cilium in the wild-type strain. In the DCDC2 or ZYG-8 overexpressing cells the cilium could not be seen. The apparent discordance between the ciliary phenotypes between C. *elegans* and mammalian neurons could be explained by the different localization of the cilium. In mammalian neurons, cilia are generally localized to the soma or the proximal part of the apical dendrite whereas in *C. elegans*, cilia are localized to the tip of the dendrite. If the altered morphology of the dendrites of the neurons in *C.elegans* is too abnormal to produce a cilium at the tip, then obviously the effect of DCDC2 overexpression on the ciliary length cannot be assessed in these cells. It is important to note that the morphological phenotype in *C.elegans* may still be cilium dependent since it can be seen only in cells that are ciliated in wild-type neurons.

In agreement with the finding in *C.elegans* mammalian cells overexpressing DCDC2 *in vitro* also showed an aberrant morphology showing an increase of branches of neurites.

Disruption of several genes associated with dyslexia, including Dcdc2, impairs the migration of developing neurons from the ventricular zone to their proper location in the cortical plate [2], [4], [5]. However, overexpression of DCDC2 did not influence the migration of newborn neurons during development [3]. Our results link DCDC2 with ciliary function but the connection between the effects on ciliary structure and signaling to the migration phenotype, if any, remains unclear. It is not known whether migrating neurons have a cilium, but it should be noted that the centrosome, which is a critical building block of cilia, plays a central role in the movement of the nucleus during neuronal migration and it is unclear whether such a role is consistent with a simultaneous expression of cilia. However, primary cilia are present in cells in proliferative zones in the brain during embryogenesis, and they have been detected again in adult brains after neuronal migration is completed [11]. The relationship between the ciliary phenotype and migration therefore remains to be clarified. The association between Kif3a and Dcdc2 is interesting since Kif3a has been shown to function in the establishment of neuronal polarity, which is highly relevant when considering neuronal migration.

Defects in cilia can cause a broad spectrum of disorders affecting multiple organs [10]. Some ciliary disorders affect the development of the central nervous system and thus can have an effect on cognitive functions. In the ciliary disease Bardet-Biedl syndrome there are language and learning deficits, which is interesting in the context of dyslexia [32]. In another ciliary disease, Joubert syndrome, one of the causative genes, *AHI1*, is required for cortical and cerebellar development [33]. Interestingly, the pericentrin gene (*PCNT*), encoding a centrosomal protein required for the assembly of the primary cilium, has recently been implicated as a new candidate gene for dyslexia [34]. Our present results raise the question of possible roles of other dyslexia candidate genes in the structure and signaling of the primary cilium.

## Materials and Methods

### Cell cultures and immunocytochemical staining

Hippocampal and cortical neuron cultures were prepared from the brains of E17 rat embryos and cultured as described previously [35] for details see File S1.

After 7 days in vitro the neuronal cells were transfected with expression constructs (File S1) using Lipofectamine 2000 (Invitrogen) according to the manufacturer's instructions with minor changes for details see File S1. 48 h after transfection the cells were fixed with 4% paraformaldehyde for 10 min.

Mouse fibroblast cell line NIH/3T3 (ATCC no. CRL-1658) or African green monkey kidney cell line COS-7 (ATCC no. CRL-1651) were used for immunocytochemical staining and microtubule pelleting assays for details on the cultures see File S1. The cells were fixed 24 h after transfection with 4% paraformaldehyde in phosphate buffered saline (PBS) for 5 min.

After fixation cells were permeabilized, blocked and incubated with primary and secondary antibodies, nuclei were stained with DAPI and the samples were mounted on microscopy slides, for details see File S1.

### Ciliary length measurements and neuronal morphology analysis

The ciliary lengths were measured from confocal immunofluorescence images (File S1) using Zeiss LSM Image Browser (Carl Zeiss). On the same images there were transfected and untransfected cells from the same cultures. The person measuring the ciliary length was blind to the status of the cells. Descriptive statistics of cilia length measurements can be found in File S1.

The effects of DCDC2 on the morphology of primary rat hippocampal cells were investigated by transfecting cells with DCDC2-V5 and GFP at the time of plating. The cells were fixed at 4 DIV, stained with neuronal marker Tuj1 and GFP, and photographed using Axioplan 2 imaging microscope. Total length of all neurites per cell as well as the number of secondary and tertiary neurites per cell (as a measure of branching) were measured by a person blind to the groups analyzed using ImageJ plug-in for Neuron J.

### *In situ* proximity ligation assays

Hippocampal neuronal cultures were grown on chamber slides and subjected to *in situ* proximity ligation assays using antibodies against DCDC2 and KIF3A according to protocol (Duolink, OLINK Bioscience) with minor adaptations. Experimental procedures can be found in more detail in File S1.

### Co-Immunoprecipitations and Western blot analysis

Whole cell protein extracts from rat hippocampal neurons and SH-SY5Y neuroblastoma cells were harvested with NP-40 buffer containing protease inhibitors (Roche). The Rat hippocampal neurons protein extracts (500–1000 ug) were incubated for 2 h at +4°C with 40 µl of protein G-sepharose slurry (GE healthcare) and 1 µg of DCDC2 antibody (sc-50728, Santa Cruz Biotechnology) in IP-T150 buffer (50 mM Tris-HCl, pH 7.4, 150 mM NaCl, 0.2% Nonidet P-40, 1 mM EDTA, and 10% glycerol). After incubation, the beads were washed three times using the IP-T150 buffer. For protein elution, beads were incubated with 1xSDS sample buffer, denatured by boiling and protein extracts were resolved in NuPage polyacrilamide 10% gels (Invitrogen). Western blot analysis was performed using the KIF3A antibody (ab11259, Abcam, 1∶2000) and anti-Rabbit as secondary antibody (sc2313 Santa Cruz Biotechnology).

### Luciferase assays

Primary embryonic rat cortical neurons were used for the luciferase assays for preparation of the cortical cultures see File S1. The cortical cells were transfected after 7 days in vitro using Lipofectamine 2000 according to manufacturer's instructions with minor changes (File S1). The cells were lysed 48 h after transfection and luciferase activity was measured using the dual luciferase reporter assay system kit (Promega) and a TD-20/20 luminometer (Turner designs) according to the manufacturer's instructions. In the luciferase experiments each construct studied was transfected on at least five parallel wells. Both the Wnt and Shh luciferase experiments were repeated independently three times.

### Microarray experiment and analysis

The total RNA samples from control and DCDC2 overexpressing neurons were prepared and hybridized according to manufacturer's protocols (Affymetrix Inc., Santa Clara, CA, USA). The arrays were scanned with GeneChip scanner 3000 7G (Affymetrix Inc.). The analyses of the microarray data were performed using the statistical software R (http://www.R-project.org). The resulting list of differentially expressed genes was subjected to GO and pathway enrichment analysis using WebGestalt (http://bioinfo.vanderbilt.edu/webgestalt/) [36]–[39]. The microarray data are MIAME compliant and the results have been submitted to the ArrayExpress database (E-MTAB-518). Some of the most differentially expressed genes were further studied by qRT-PCR, primers can be found in File S1. Experimental procedures can be found in more detail in File S1.

### *C. elegans* expression constructs, transgenic strains and microscope analyses

About 700 bp of the proximal gcy-32 gene promoter were cloned into the MCS of the basic GFP vector pPD95.75, upstream of the translational start site of the GFP gene. cDNA's of the *C. elegans* gene zyg-8 (2.4 kb) and of the human gene DCDC2 (1.4 kb) were then introduced into the above described gcy-32::gfp expression construct by replacing the GFP gene coding sequence, resulting in gcy-32::zyg-8 and gcy-32::DCDC2 expression constructs, respectively. The gcy-32 gene promoter drives expression in the *C. elegans* neurons AQR and PQR (ciliated) and URX (non-ciliated) [40]. Transgenic worm lines were generated following standard microinjection procedures [41]: “*control lines*” containing a gcy-32::gfp plus empty vector DNA transgene, “*zyg-8 lines*” containing a gcy-32::gfp plus gcy-32::zyg-8 transgene, and “*DCDC2 lines*” containing a gcy-32::gfp plus gcy-32::DCDC2 transgene. 100 ng/µl of the relevant constructs were injected. At least three independent transgenic lines per construct were generated and analyzed. Two independent transgenic lines per construct were used for quantitation, where at least 25 young adult animals were analyzed for each genotype and for all three neurons, respectively (see Figure 5). A Zeiss LSM 510 META confocal microscope setup was used for taking pictures for details on microscopy see File S1.

## Supporting Information

File S1 **More detailed information on material and methods.**
(DOCX)

Figure S1 **DCDC2-V5 is pelleted together with microtubules.** Polymerized microtubules were incubated with cell lysates from NIH/3T3 cells overexpressing DCDC2-V5, DCDC2-Del1-V5, DCDC2-Del2-V5 or LacZ-V5. Microtubules pellet when centrifugated at 100 000×g, and any proteins that bind to them will pellet with them. DCDC2-V5 (A and B), DCDC2-Del1-V5 (A) and DCDC2-Del2-V5 (A) all pelleted with microtubules, but not in control samples without microtubules. Control protein LacZ-V5 could only be detected in the supernatant and did not pellet with or without microtubules (B). A control western blot with anti-tubulin antibody verified that tubulin had pelleted (C).
(TIF)

Figure S2 **Transfected cells in rat embryonal primary hippocampal cultures were predominantly neuronal.** Confocal images of rat primary neurons transfected with DCDC2-V5 and immunolabeled with antibodies againsts V5 epitope (B,E) and neuronal markers Tuj-1 (A) or NeuN (D). Panels C and F show merged images and scale bars indicate 10 µm.
(TIF)

Figure S3 **DCDC2-V5 localizes on microtubule networks in non-ciliated cells and in primary cilia in ciliated cells.** COS-7 cell transfected with DCDC2-GFP (A). Image was taken with a fluorescence microscope. Primary rat hippocampal neuron transfected with DCDC2-V5/His and labeled with anti-V5 (C), neuronal ciliary marker anti-Ac3 (B) and nuclear stain DAPI in blue (D). Localization of DCDC2 in the primary cilium in mouse fibroblast cell line NIH/3T3. The cells were transfected with DCDC2-V5 and immunolabeled with antibodies against the ciliary marker acetylated tubulin (E) and anti-V5 (F). Nuclei were labeled with DAPI and are seen in blue in the merged image (G). The ciliary localization in NIH/3T3 cells was further confirmed by staining DCDC2-V5 transfected cells with DNAL4 a centriolar marker that stains the centriole at the base of the cilium (H–J). Confocal images. Scale bars indicate 10 µm.
(TIF)

Figure S4 **Deletion constructs of DCDC2 are not dominant negative for the ciliary localization of full-length DCDC2.** Rat primary hippocampal neurons cotransfected with DCDC2-GFP and DCDC2 deletion constructs lacking either of the doublecortin domains; DCDC2-Del1-V5 (A–C) or DCDC2-Del2-V5 (D–F). The cells were immunolabeled with antibodies against GFP and V5. Nuclei were labeled blue with DAPI. Confocal images. Scale bars indicate 10 µm.
(TIF)

Figure S5 **Overexpression of DCDC2 increases ciliary length significantly in NIH/3T3 cells.** Ciliary length in NIH/3T3 cells transfected with different constructs. The error bars are 95% confidence intervals of the relative ciliary length. Each transfected construct was compared to untransfected cells from the same cultures with two tailed Student's t-test not assuming equal variances. The difference in ciliary length between DCDC2 and untransfected cells was significant (* p = 2,6×10^−5^), but all the other comparisons were not statistically significant. The ciliary length measurements were also done in primary rat hippocampal cells (Fig. 2B).
(TIF)

Figure S6 **Verification of the specificity of the DCDC2 antibody (sc-50728 Santa Cruz Biotechnology).** Detection of Dcdc2 after immunoprecipitation with DCDC2 antibody (blot A lane 5). Detection of DCDC2 in SHSY5-cells (blot A lane 2) and rat primary cortical neurons (blot B lane 1) after overexpression of DCDC2. Immunocytofluorescent staining showing that Dcdc2 antibody recognizes the overexpressed form of DCDC2-V5 by co-staining with the tag marker V5 (C–E).
(TIF)

Figure S7 **The increase in canonical Wnt signaling after Dcdc2 knock-down is inhibited by simultaneously expressing DCDC2-V5.** Knocking-down Dcdc2 with shRNA leads to increased Wnt signaling as measured by a luciferase reporter assay, this increase can be blocked by simultaneously over-expressing DCDC2-V5 (* p<0,05, ANOVA, followed by T-test.)
(TIF)

Figure S8 **Gene ontology analysis of the microarray data of differentially expressed genes after DCDC2 overexpression.** There is enrichment in GO terms of the cellular component in microtubule cytoskeleton (adjusted p-value 2.49×10^−5^) as well as in biological processes related to cell cycle.
(TIF)

Figure S9 **Differential expression of five genes upregulated upon DCDC2 overexpression in hippocampal neurons.** The mRNA expression levels were measured by qRT-PCR in triplicates from eight technical replicates. Relative expression levels of the genes in the DCDC2 overexpressing neurons were compared to expression levels in control transfected neurons using Ppia as endogenous control and using ΔΔCt method (mean fold-change ± SEM). Similar results were obtained when Hprt was used as endogenous control. Significance was tested using Student's t-test using ΔCt values (***p<0.005, ** p<0.01 ja *p<0.05).
(TIF)

Figure S10 **Overexpression of DCDC2 leads to increased branching in rat hippocampal neurons.** Cells were transfected with either DCDC2-V5 and GFP or GFP only before plating. The morphology was analyzed by measuring length and branching of neurites using NeuronJ plug-in of ImageJ. Branching (A) but not total length (B) was significantly changed after overexpression of DCDC2 (*p<0,05, T-test using Welch correction, DCDC2-V5 n = 44, GFP n = 61).
(TIF)
