# Supplementary material for: Increased Expression of the Dyslexia Candidate Gene DCDC2 Affects Length and Signaling of Primary Cilia in Neurons
Source: PLoS One. 2011 Jun 16;6(6):e20580. doi: 10.1371/journal.pone.0020580 (PMC3116825; doi:10.1371/journal.pone.0020580)
Supplement: File S1 — More detailed information on material and methods. (DOCX) [file pone.0020580.s001.docx]

**Increased expression of the dyslexia candidate gene DCDC2 affects length and signaling of primary cilia in neurons**

## Satu Massinen, Marie-Estelle Hokkanen, Hans Matsson, Kristiina Tammimies, Isabel Tapia-Páez, Vanina Dahlström-Heuser, Juha Kuja-Panula, Jan Burghoorn, Kristian E. Jeppsson, Peter Swoboda, Myriam Peyrard-Janvid, Rune Toftgård, Eero Castrén, Juha Kere

***Supplementary information methods***

### Preparation of expression constructs

The full length DCDC2 constructs were cloned into pcDNA3.1/V5-His-TOPO and pcDNA3.1/NT-GFP-TOPO vectors (Invitrogen) using the TOPO cloning strategy according to the manufacturer’s instructions. The insert in the DCDC2-V5 plasmid is bases 261-1730 of *DCDC2* cDNA (NM_016356.3). The V5 epitope tag is in the C-terminus of the resulting fusion protein. The insert in DCDC2-GFP construct is bases from 306 to 1733 of the *DCDC2* cDNA. The GFP tag is in the N-terminus of the resulting fusion protein. The sequence of the vectors was confirmed by DNA sequencing.

The deletion constructs were prepared using the DCDC2-V5 plasmid as a template. Two PCR fragments were first amplified from each side of the deletion. The DCDC2-Del1-V5 construct lacks the first doublecortin domain (residues 12 - 100 in NP_057440.2, nucleotides 336 - 602 in NM_016356.3). The DCDC2-Del2-V5 construct lacks the second doublecortin domain (residues 134-221 in NP_057440.2, nucleotides 702-965 in NM_016356.3). The 5’ portions of the deletion constructs were amplified using a shared forward primer that anneals upstream of the initiation codon and deletion-specific reverse primers. The 3’ portions were amplified using deletion specific forward primers with a shared reverse primer that anneals at the 5’ side of the stop codon. The primary PCR products were mixed and used as templates in secondary PCRs with the shared forward and reverse primers. The PCR fragments containing deletions were directly cloned into the pcDNA-V5-His vector using the TOPO TA cloning kit (Invitrogen). The sequence of the vectors was confirmed by DNA sequencing.

The Dcdc2-shRNA and control-shRNA constructs were cloned into mU6pro vector [1]. The vector was digested with restriction enzymes Bbs1 (New England Biolabs) and Xba1 (Fermentas) and annealed oligomers containing the required inserts were ligated into the vector. The resulting shRNA molecules are identical to the ones used in previous Dcdc2 knockdown experiments [1]. The sequence of all constructs was confirmed by DNA sequencing. The control vector used in microtubule pelleting assays, pcDNA3.1/V5-His-TOPO/LacZ was purchased from Invitrogen.

In the ciliary length measurements the overexression constructs and the shRNA plasmids were cotransfected with pEGFP-F (Clontech) control vector in order to visualize the cotransfected cells.

**Preparation of primary neuronal cultures**

Preparation of hippocampal cultures was done briefly as follows, the hippocampi from E17 Wistar rat embryos were dissected, the meninges removed and the neurons dissociated in single-cell suspension with papain (0.5 mg/ml) digestion and mechanical trituration. The cells were centrifuged, suspended in DMEM containing Glutamax I and supplemented with 10% fetal bovine serum (FBS), 100 U/ml penicillin, and 100 μg/ml streptomycin (DMEM medium; Gibco BRL). 100000 cells/well on 12-well plates were plated onto glass coverslips coated with 0.5 mg/ml poly-L-ornithine (Sigma) and 10 mg/ml laminin (Invitrogen.). The cells were cultured in Neurobasal medium (Gibco) supplemented with B27 (Gibco) , penicillin-streptomycin (Euroclone) and L-glutamine (Euroclone) at 37 ºC in 5% CO_2_.

Cortical neuron cultures were prepared from the brains of E17 rat fetuses. The forebrain cortices were dissected, the meninges removed and the neurons dissociated in single-cell suspension by mechanical trituration using a 20 G needle. The cells were centrifuged, resuspended in DMEM supplemented with 10% fetal bovine serum (FBS), l-glutamate, 100 U/ml penicillin, and 100 μg/ml streptomycin (DMEM medium; Gibco BRL). Cells were plated on 10 cm bacterial plates for 30 min and then carefully recollected and centrifuged. The cell pellets were then resuspended in Neurobasal medium. 600000 cells/well were plated on 24-well plates coated with 0,001% poly-L-lysine (Sigma). Cells were cultured in Neurobasal medium (Gibco) supplemented with B27 (Gibco), penicillin-streptomycin (Euroclone) and L-glutamine (Euroclone) at 37 ºC in 5% CO_2_.

**Cell line cultures**

Mouse fibroblast cell line NIH/3T3 or African green monkey kidney cell line COS-7 were grown on glass coverslips on 24 well plates in DMEM medium containing Glutamax I and supplemented with 10% fetal bovine serum, 100 U/ml penicillin, and 100 μg/ml streptomycin.

For the microtubule pelleting assays, NIH/3T3 cells were grown on culture dishes (10cm in diameter).

**Transfections of cell line cultures and primary neuronal cultures**

Transfections of hippocampal neuronal cultures for immunocytochemical stainings were done according to manufacturers protocol with the following details Lipofectamine (4 µl) and a total of 2 µg of DNA/well were used. In cotransfection experiments an equal amount of constructs was used. 30 min before transfection the medium was changed to antibiotics free medium and 6 h after transfection the medium was changed back to conditioned medium.

The transfection of cortical cells for luciferase assays was done as for the hippocampal neurons except that the amount of lipofectamine and DNA was double compared to the manufacturers protocol (4 µl lipofectamine and 2 µg total DNA/24-well). The cells were transfected with the respective plasmid constructs, a TOPflash firefly luciferase construct for measuring Wnt signaling or Gli firefly luciferase construct for measuring Shh signaling and a constitutively active renilla luciferase construct (Promega) for normalization of transfection efficiency. The TOPflash firefly luciferase construct contains 3 TCF binding sites and was kindly provided by Doctor Seppo Vainio. The Gli firefly luciferase construct contains 8 Gli3 binding sites and was kindly provided by Dr Jussi Taipale. For cotransfections of the different constructs, a ratio of 4:4:1 (respective plasmid: TOPflash/Gli-luciferase:renilla luciferase) was used.

For the immunocytochemical staining of NIH/3T3 or COS-7 cells, transfections of the expression constructs were carried out with FuGENE 6 transfection reagent (Roche Applied Science) according to the manufacturer’s instructions for details see above. A total of 500 ng of DNA and 1,5 µl of transfection reagent per well was used. For NIH/3T3 cells, 5 h after transfection the medium was changed to serum free medium.

For the microtubule pelleting assays, NIH/3T3 cells were transfected with Lipofectamine (Invirtogen) according to the manufacturer’s instructions using 8µg of plasmid DNA and 100µl of Lipofectamine per dish.

**Immunocytochemical stainings**

Cells were permeabilized with 0,1% Triton X-100 in PBS (for NIH/3T3 cells and COS-7 cells) or with ice-cold methanol (for neuronal cells). The cells were incubated first in 3% bovine serum albumin (BSA) in PBS for 30 min to block unspecific binding of the antibodies. Subsequently the cells were incubated with primary antibodies for 45 min followed by three washing steps with PBS and staining with secondary antibodies for 45 min. Finally the nuclei were stained with 4’,6-diamino-2-phenylindole (DAPI, Sigma-Aldrich) and the cells were mounted on glass slides with ProLong Gold antifade reagent (Invitrogen).

**Antibodies used in this study**

The primary antibodies used in this study were anti-Ac3 produced in rabbit (Adenylyl cyclase III) (sc-588, Santa Cruz Biotechnology), anti-V5 produced in rabbit (NB-600-381, Novus Biologicals), anti-V5 produced in mouse (R960, Invitrogen), anti-gamma-tubulin produced in rabbit (sc-10732, Santa Cruz Biotechnology), anti-acetylated tubulin produced in mouse (T7451, Sigma), anti-DNAL4 (HPA003647, Atlas antibodies), anti-GFP produced in rabbit (ab290, Abcam), anti Tuj1 produced in rabbit (MMS-435P, Biosite) and anti NeuN (MAB377, Chemicon), anti-KIF3A, produced in rabbit (ab11259, Abcam), anti DCDC2 produced in goat (sc-50728, Santa Cruz Biotechnology).

The secondary antibodies used in this study were Alexa Fluor 488 goat anti-rabbit IgG (H + L), Alexa Fluor Alexa Fluor 555 donkey anti-rabbit IgG (H + L), Alexa Fluor 555 goat anti-mouse IgG (H + L), Alexa Fluor 488 goat anti-mouse IgG (H + L) (Invitrogen) and Cy3-conjugated goat anti-mouse IgG (Jackson ImmunoResearch).

**Microscopy**

[The confocal immunofluorescence images were acquired with](http://www.biocompare.com/prorev.asp?profrevid=471) Meta LSM 510 confocal imaging system and Axiovert 200 M microscope (Carl Zeiss) using 63× NA 1.4 or 40× NA 1.2 objective and multichannel scanning in frame mode. Settings for the blue channel used for imaging DAPI staining were: excitation wavelength 405 nm with emission filter BP 420-480. Settings for the green channel used for imaging EGFP or the Alexa Fluor 488 secondary antibody conjugates were: excitation wavelength 488 nm with emission filter BP 505-530. Settings for the red channel used for imaging the Cy3 or Alexa Fluor 555 conjugated secondary antibody conjugates were: excitation wavelength 543 nm with emission filter BP 560-615, BP 575-615, BP 585-615 or LP575.

The pictures for figure 6 were taken with ZEISS LSM 510 META confocal microscope. Serial images through the entire worm were acquired along the z-axis in the 488-nm channel. 3D images were then reconstructed from the stacks of image files using Zeiss LSM software. The resulting images were then processed with Adobe Photoshop software.

Supplementary Fig. 3a was acquired using Axioplan 2 imaging microscope (Carl Zeiss) using Plan-Neofluar 100X NA 1.3 objective and Filterset 16 (488016-0000; excitation BP485/20, beamsplitter FT510 and emission LP515). Image acquisition software used was AxioVision 3.0 (Carl Zeiss).

***In situ* proximity ligation assays**

Hippocampal neuronal cultures were plated onto each well in Lab-Tek II chamber slides (Nunc) coated with 0.001% poly-L-ornithine (Sigma) and 10 mg/ml laminin (Invitrogen). The cells were grown 7 days in vitro after which they were washed in cold PBS, fixed in ice-cold 70% ethanol for one hour and air-dried before storage at -20˚C.

The chamber wells were separated from the glass and a hydrophobic barrier between individual wells was made using a ImmEdge pen (Vector Laboratories, H-4000). Blocking, antibody hybridizations, proximity ligations [3-5] and detections were performed according to protocol (Duolink IQ, OLINK Bioscience) with minor adaptations. Briefly, Rabbit anti KIF3A, (ab11259, Abcam) at 1μg/ml and Goat anti DCDC2 (sc-50728, Santa Cruz Biotechnology) at 1μg/ml were incubated at 4ºC overnight for detection of protein-protein interactions in rat hippocampal neurons. We applied combinations of anti-Goat PLUS and anti-Rabbit MINUS PLA probes for 45 minutes or one hour at 37ºC. Rabbit anti KIF3A, (ab11259, Abcam) at 1μg/ml and Goat anti DCDC2 (sc-50728, Santa Cruz Biotechnology) at 1μg/ml. Subsequent hybridizations, ligations and detections using DuoLink™ 100 Detection kit 563 (OLINK Bioscience) were performed. DuoLink™ 100 Detection kit 563 includes a Tye 563 fluorophore with excitation at 557 nm and emission at 563 nm and Hoechst 33432 nuclear dye. Phosphate buffered saline with 0.05 % Tween-20 (PBS-T) was used for washing. For staining of cilia we used Rabbit anti Adenylyl cyclase III, Ac3, (sc-588, Santa Cruz Biotechnology) and Fluorescein isothiocyanate (FITC) conjugated Goat anti Rabbit (H&L) (ab6717, Abcam) at a 1:100 dilution in PBT-T. Preparations were mounted in DuoLink™ Mounting Medium, (OLINK Bioscience) under cover glass and sealed with nail polish. Assays were photographed using Olympus IX71 inverted fluorescence microscope with oil immersion 100x lens. Filter settings in nanometers for emission, excitation and mirror for the Hoechst 33432 were 350/50, 460/50, 400 LP; for FITC 480/40, 535/50, 505 LP and for the red channel 565/30, 620/60, 585 LP, respectively. Brightness and contrast of photos from the microscope were enhanced for presentation using the ImageJ 1.38x software and figures were assembled in Adobe Illustrator CS2 software.

**Microarray experiment and analysis**

Total RNA from control and DCDC2 overexpressing hippocampal neurons were isolated with Mini RNA isolation kit II (Zymo Research) and the concentrations were measured by a UV spectrophotometer and the quality determined using the RNA Nano LabChip kit on the Agilent 2100 Bioanalyzer (Agilent technologies, DE, USA).The samples were prepared and hybridized according to manufacturers protocols (Affymetrix Inc., Santa Clara, CA, USA). The arrays were scanned with GeneChip scanner 3000 7G (Affymetrix Inc.). The Rat Gene 1.0 ST array constitutes more than 27,000 gene-level probe sets.

The analyses of the microarray data were performed using the statistical software R (http://www.R-project.org), by implementing the packages Affy and Limma [6, 7]. For the annotation of the probe sets in the Rat Gene 1.0 ST array, we used the cdf package RaGene10stv1_Rn_ENTREZG version 11 downloaded from <http://brainarray.mbni.med.umich.edu/Brainarray/default.asp>. Background signals subtraction, normalization and Log2 expression values were calculated using the robust multiarray average method (RMA) (8). A linear model was fitted to the expression data for each probe using the least squares method. Contrasts were specified to make pair-wise comparison between the control and DCDC2 overexpression groups, and coefficients and estimated standard errors were computed based on the fitted linear models. The estimated coefficients and standard errors were used to compute moderated t-statistics and log-odds of differential expression (B-values), using empirical Bayes shrinkage of the standard errors towards a common value [7].

**GO enrichment analysis**

GO enrichment analysis was done using our gene list (Table 1) and Rat Gene st V1.0 as background. To test if any gene ontology terms were enriched in our differentially expressed genes list from the microarray experiment, we used the WebGestalt bioinformatics resources [9, 10]. Adjusted p-value 0,05 was used as a threshold in the functional annotation clustering.

#### Quantitative real-time PCR

For cDNA synthesis 0,5 µg of total RNA were used as template using the SuperScript® III First-Strand Synthesis SuperMix kit (11752-250, Invitrogen). The PCR assay was performed using Fast SYBR® Green PCR Master Mix (4385612, Applied Biosystems) in a total volume of 10 μl, containing 1:5 diluted cDNA templates. The reactions were amplified using 7500 Fast Real-Time PCR system (Applied Biosystems). A dissociation stage was added to confirm primer specificity. Relative expression of the different gene transcripts were calculated by using the ΔΔCt method and converted to the relative expression ratio (2^-^ ^ΔΔCt^). All data were normalized to the endogenous reference genes peptidylprolyl isomerase A (Ppia) and hypoxanthine-guanine phosphoribosyltransferase (Hprt) expression. Student’s t-test were performed using the ΔCt values, sample variances were tested to be equal. Primer sequences are in the supplementary table S2.

### Statistical analyses

In the ciliary length measurements the cilium in each group of transfected cells was compared to that of untransfected cells from the same cultures. Ciliary length can be assumed to be normally distributed and the length histograms for each group appeared to be in agreement with this assumption (data not shown). The ciliary lengths were analyzed with two-tailed student’s t-test not assuming equal variances. For Fig. 2b, the 95% confidence interval for the ratio of mean ciliary length in transfected cells versus untransfected cells was calculated by the method of EC Fieller [11]. In the luciferase assays, the ratio of reporter luciferase to control luciferase was normalized to the mean value of the shRNA-control transfected cells. The groups transfected with different DCDC2 or control constructs were compared to each other using one-way ANOVA followed by Tukey's Multiple Comparison Test which showed that statistically significant results were found only when comparing DCDC2 overexpression against other groups in the Shh assay and when comparing shRNA-Dcdc2 to other groups in the Wnt assay.

**Table S1.** Descriptive statistics of ciliary length measurements.

|  |  | **Transfected** | | **untransfected** | |
| --- | --- | --- | --- | --- | --- |
| **Cells** | **transfection** | **n** | **mean length (µm) ± SD** | **n** | **mean length (µm) ± SD** |
| Rat embryonic hippocampal neurons | DCDC2-V5 | 52 | 5.3 ± 2.7 | 102 | 2.6 ± 1.0 |
|  | DCDC2-Del1-V5 | 54 | 2.5 ± 1.1 | 114 | 2.6 ± 1.1 |
|  | DCDC2-Del2-V5 | 54 | 2.8 ± 1.2 | 142 | 2.7 ± 0.9 |
|  | DCDC2-shRNA | 136 | 2.6 ± 1.2 | 545 | 2.5 ± 1.0 |
|  | control-shRNA | 56 | 2.6 ± 0.8 | 236 | 2.4 ± 0.7 |
|  | GFP | 44 | 2.1 ± 0.5 | 185 | 2.2 ± 0.6 |
| NIH/3T3 | DCDC2-V5 | 53 | 6.1 ± 3.2 | 410 | 4.1 ± 2.0 |
|  | DCDC2-Del1-V5 | 52 | 3.6 ± 1.7 | 336 | 3.4 ± 1.5 |
|  | DCDC2-Del2-V5 | 49 | 3.5 ± 2.2 | 539 | 3.5 ± 1.6 |
|  | DCDC2-shRNA | 56 | 2.9 ± 1.0 | 162 | 2.9 ± 1.2 |
|  | control-shRNA | 56 | 3.0 ± 1.4 | 160 | 3.3 ± 1.4 |
|  | GFP | 51 | 2.9 ± 1.2 | 335 | 3.2 ± 1.2 |

**Table S2.** Primers used for qPCR confirmation of microarray results.

| **Gene name** | **Forward primer** | **Reverse primer** |
| --- | --- | --- |
| Pdgfra | ACGTTCAAGACCAGCGAGTT | CGATCGTTTCTCCTGCCTTA |
| Kif2c | CGGTGAACTTGGAGAAATCC | TTCGGTCGTAAGGGAAGAAG |
| Kif4 | TGACTTTGAATTTACTCTGAAAGTGTC | CCCGGATATTTATTTGAGATGC |
| Hhip | GCACTTCCACTCCTCTGCAA | AGCAAAATTCATCAGCAGTTGT |
| Cdc2 | CCTGGACTCTATCCCTCCTG | CTCGTCGGGAGTGACAAAAC |
| Ppia | AGGGTTCCTCCTTTCACAGA | ATCCAGCCATTCAGTCTTGG |
| Hprt | CTCATGGACTGATTATGGACAGGAC | GCAGGTCAGCAAAGAACTTATAGCC |

***Supplementary references***

1. Yu JY, DeRuiter SL & Turner DL (2002) RNA interference by expression of short-interfering RNAs and hairpin RNAs in mammalian cells. Proc Natl Acad Sci U S A 99: 6047-6052.

2. Meng H, Smith SD, Hager K, Held M, Liu J et al (2005) DCDC2 is associated with reading disability and modulates neuronal development in the brain. Proc Natl Acad Sci U S A 102: 17053-17058.

3.Fredriksson S, Gullberg M, Jarvis J, Olsson C, Pietras K et al (2002) Protein detection using proximity-dependent DNA ligation assays. Nat Biotechnol 20: 473-477.

4. Soderberg O, Gullberg M, Jarvius M, Ridderstrale K, Leuchowius KJ et al (2006) Direct observation of individual endogenous protein complexes in situ by proximity ligation. Nat Methods 3: 995-1000.

5. Yu S, Avery L, Baude E & Garbers DL (1997) Guanylyl cyclase expression in specific sensory neurons: A new family of chemosensory receptors. Proc Natl Acad Sci U S A 94: 3384-3387.

6. Gautier L, Cope L, Bolstad BM & Irizarry RA (2004) Affy--analysis of affymetrix GeneChip data at the probe level. Bioinformatics 20: 307-315.

7. Smyth GK (2004) Linear models and empirical bayes methods for assessing differential expression in microarray experiments. Stat Appl Genet Mol Biol 3: Article3.

8. Irizarry RA, Hobbs B, Collin F, Beazer, Barclay YD, Antonellis KJ et al (2003) Exploration, normalization, and summaries of high density oligonucleotide array probe level data. Biostatistics 4: 249-264.

9. Duncan DT, Prodduturi N & Zhang B (2010) WebGestalt2: An updated and expanded version of the web-based gene set analysis toolkit. . BMC Bioinformatics 11: 10.

10. Zhang B, Kirov SA & Snoddy JR (2005) WebGestalt: An integrated system for exploring gene sets in various biological contexts. Nucleic Acids Res 33: W741-748.

11. Fieller EC (1940) The biological standardization of insulin. Supplement to the Journal of the Royal Statistical Society 7: 1-64.
