# Supplementary figures and images for: Increased Expression of the Dyslexia Candidate Gene DCDC2 Affects Length and Signaling of Primary Cilia in Neurons
Source: PLoS One. 2011 Jun 16;6(6):e20580. doi: 10.1371/journal.pone.0020580 (PMC3116825; doi:10.1371/journal.pone.0020580)

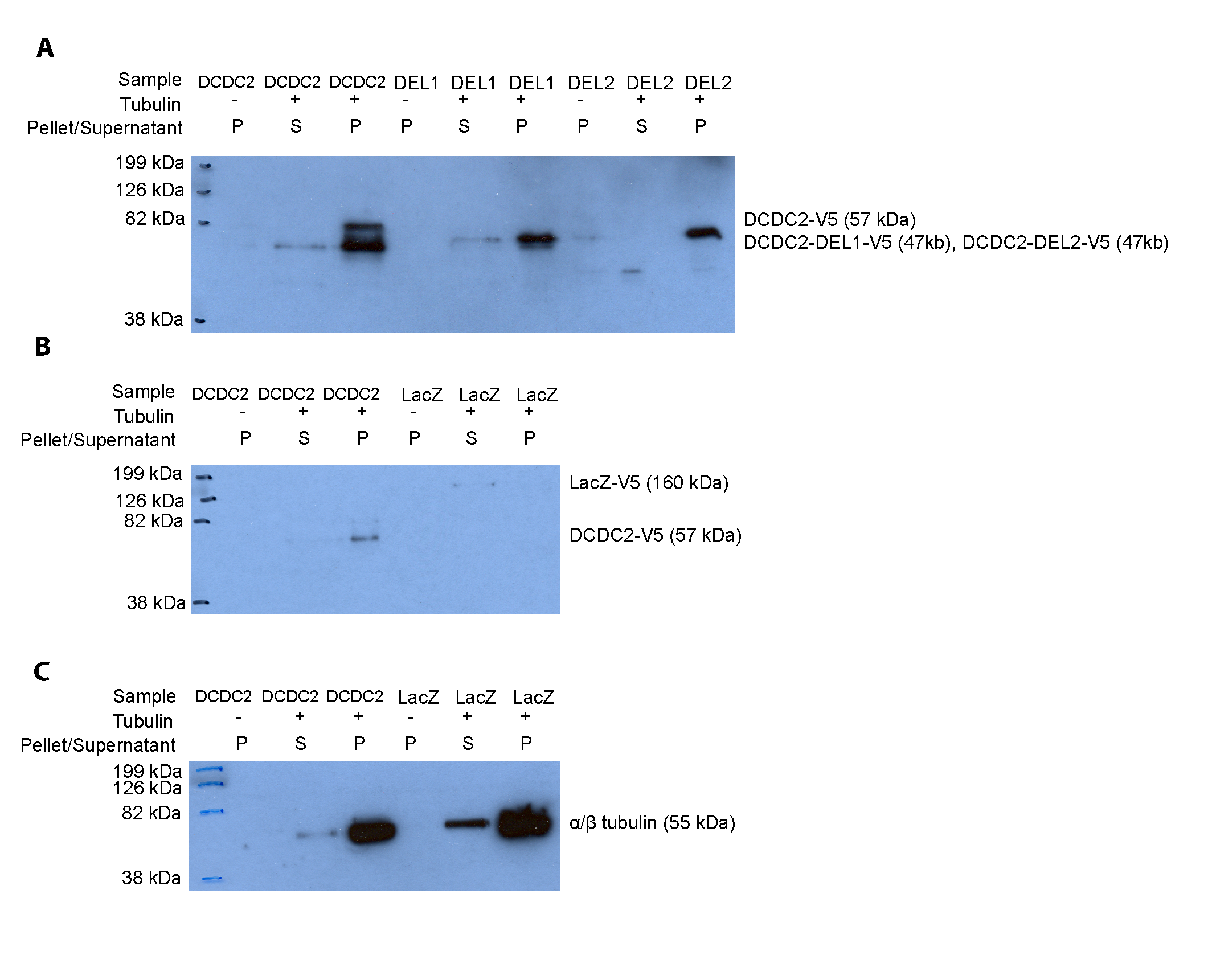

Supplement: Figure S1 — DCDC2-V5 is pelleted together with microtubules. Polymerized microtubules were incubated with cell lysates from NIH/3T3 cells overexpressing DCDC2-V5, DCDC2-Del1-V5, DCDC2-Del2-V5 or LacZ-V5. Microtubules pellet when centrifugated at 100 000×g, and any proteins that bind to them will pellet with them. DCDC2-V5 (A and B), DCDC2-Del1-V5 (A) and DCDC2-Del2-V5 (A) all pelleted with microtubules, but not in control samples without microtubules. Control protein LacZ-V5 could only be detected in the supernatant and did not pellet with or without microtubules (B). A control western blot with anti-tubulin antibody verified that tubulin had pelleted (C). (TIF) [file pone.0020580.s002.tif]

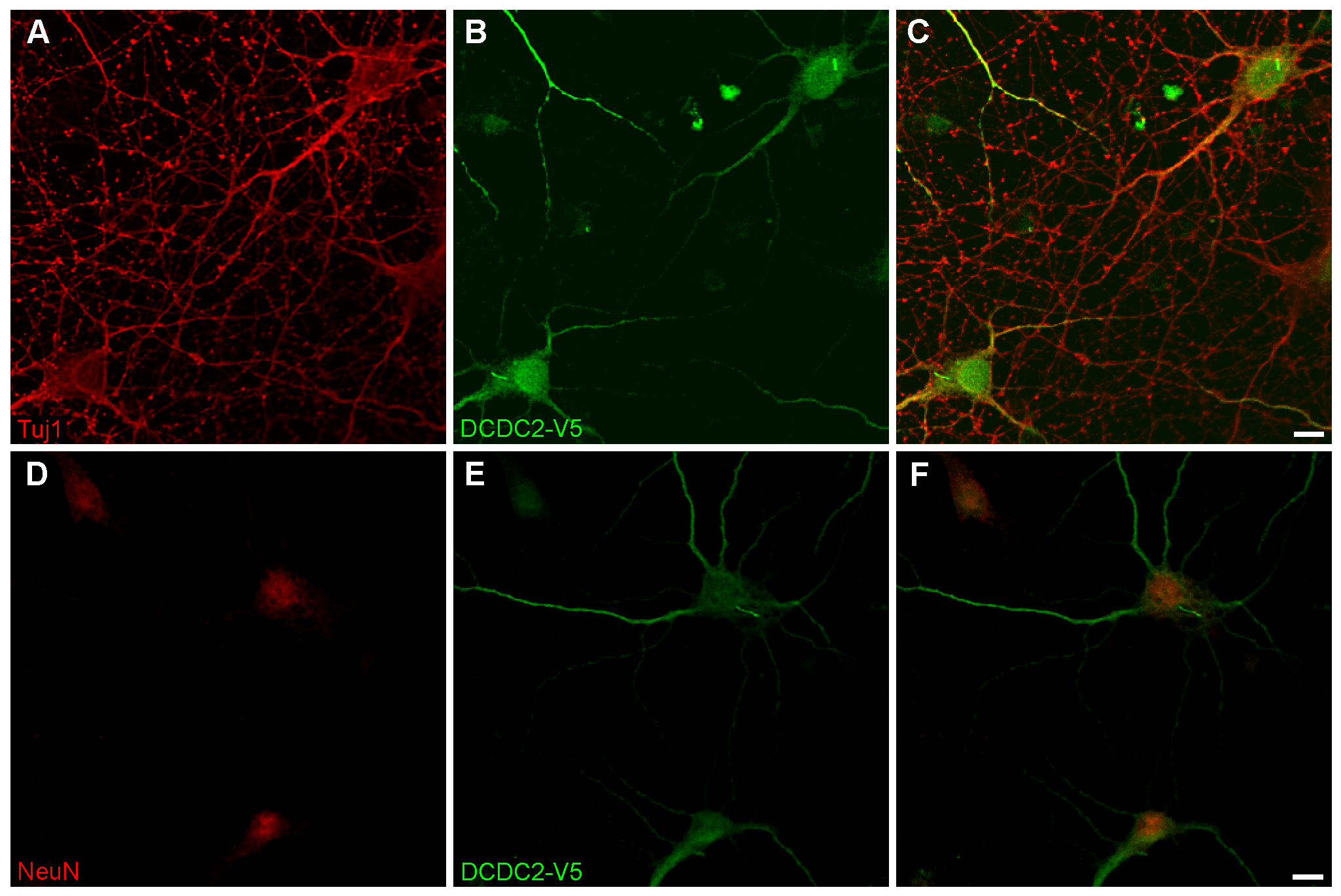

Supplement: Figure S2 — Transfected cells in rat embryonal primary hippocampal cultures were predominantly neuronal. Confocal images of rat primary neurons transfected with DCDC2-V5 and immunolabeled with antibodies againsts V5 epitope (B,E) and neuronal markers Tuj-1 (A) or NeuN (D). Panels C and F show merged images and scale bars indicate 10 µm. (TIF) [file pone.0020580.s003.tif]

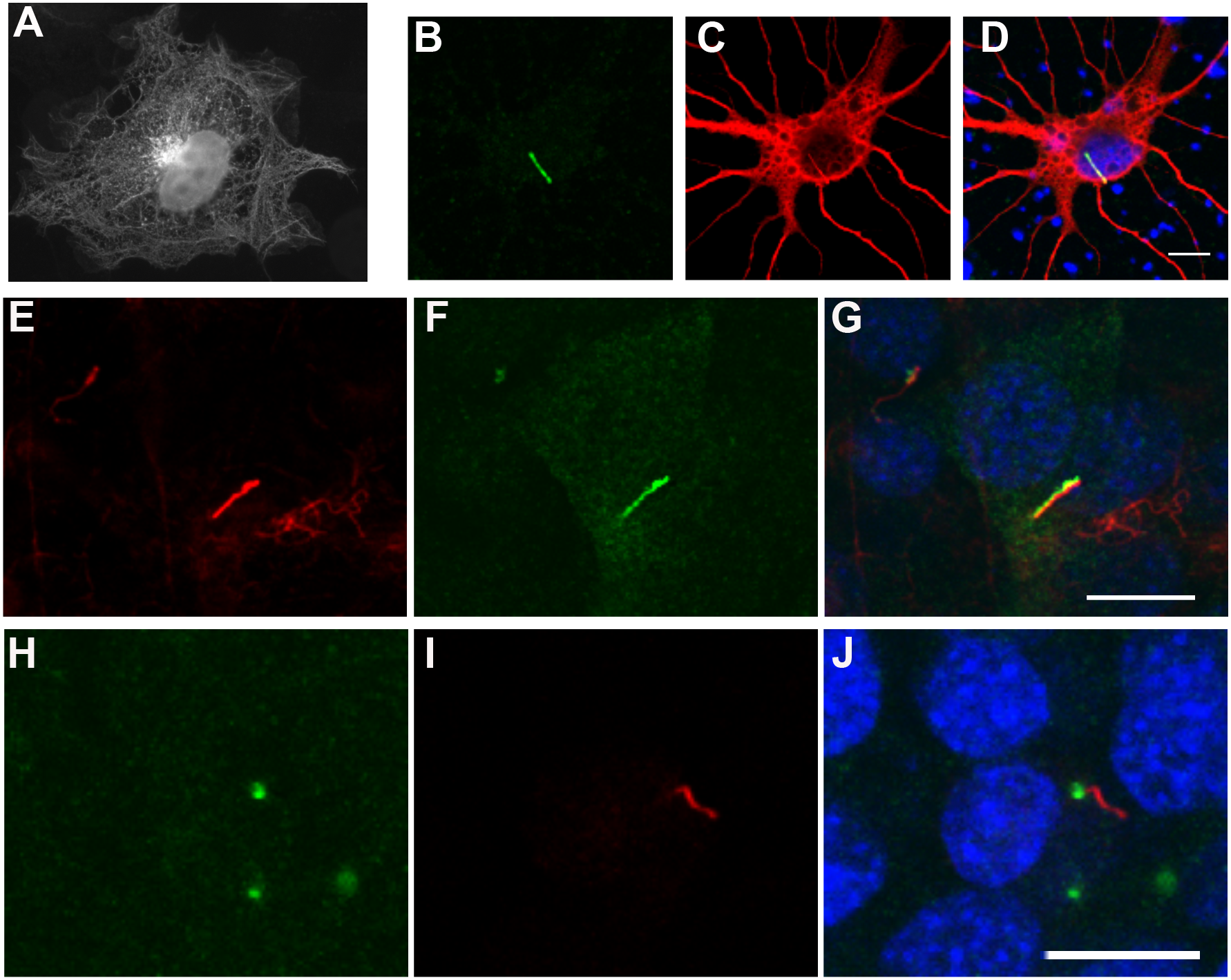

Supplement: Figure S3 — DCDC2-V5 localizes on microtubule networks in non-ciliated cells and in primary cilia in ciliated cells. COS-7 cell transfected with DCDC2-GFP (A). Image was taken with a fluorescence microscope. Primary rat hippocampal neuron transfected with DCDC2-V5/His and labeled with anti-V5 (C), neuronal ciliary marker anti-Ac3 (B) and nuclear stain DAPI in blue (D). Localization of DCDC2 in the primary cilium in mouse fibroblast cell line NIH/3T3. The cells were transfected with DCDC2-V5 and immunolabeled with antibodies against the ciliary marker acetylated tubulin (E) and anti-V5 (F). Nuclei were labeled with DAPI and are seen in blue in the merged image (G). The ciliary localization in NIH/3T3 cells was further confirmed by staining DCDC2-V5 transfected cells with DNAL4 a centriolar marker that stains the centriole at the base of the cilium (H–J). Confocal images. Scale bars indicate 10 µm. (TIF) [file pone.0020580.s004.tif]

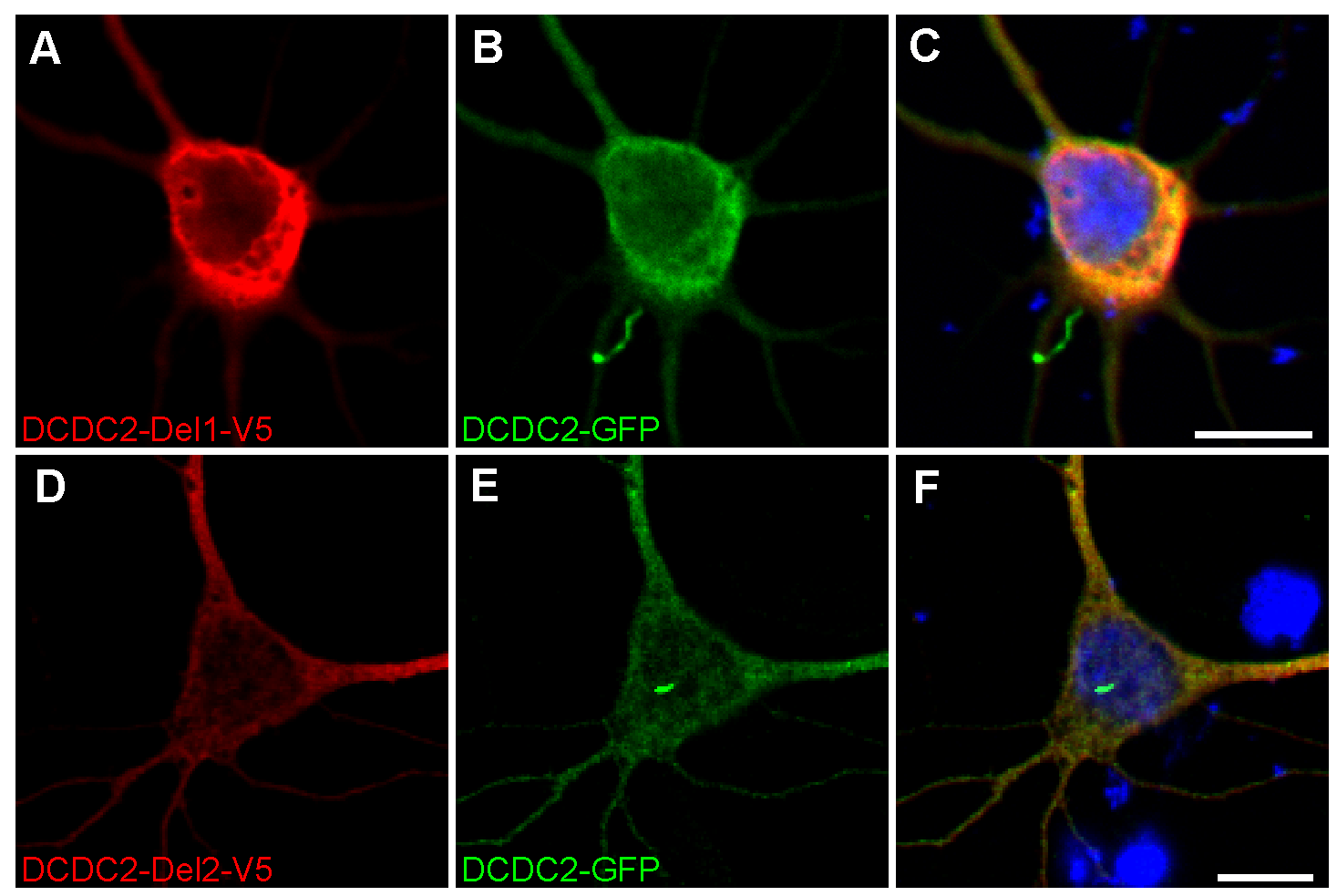

Supplement: Figure S4 — Deletion constructs of DCDC2 are not dominant negative for the ciliary localization of full-length DCDC2. Rat primary hippocampal neurons cotransfected with DCDC2-GFP and DCDC2 deletion constructs lacking either of the doublecortin domains; DCDC2-Del1-V5 (A–C) or DCDC2-Del2-V5 (D–F). The cells were immunolabeled with antibodies against GFP and V5. Nuclei were labeled blue with DAPI. Confocal images. Scale bars indicate 10 µm. (TIF) [file pone.0020580.s005.tif]

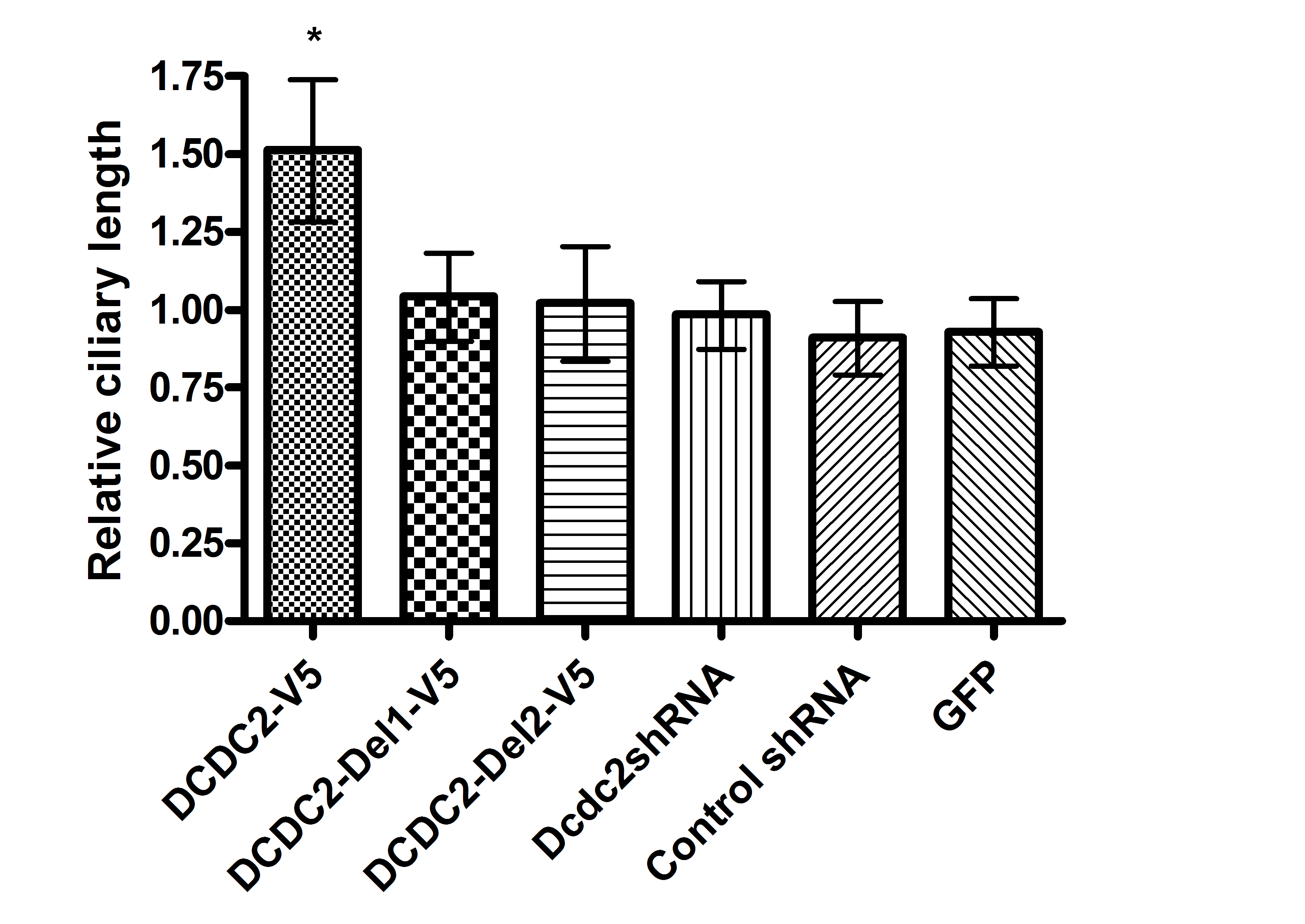

Supplement: Figure S5 — Overexpression of DCDC2 increases ciliary length significantly in NIH/3T3 cells. Ciliary length in NIH/3T3 cells transfected with different constructs. The error bars are 95% confidence intervals of the relative ciliary length. Each transfected construct was compared to untransfected cells from the same cultures with two tailed Student's t-test not assuming equal variances. The difference in ciliary length between DCDC2 and untransfected cells was significant (* p = 2,6×10^−5^), but all the other comparisons were not statistically significant. The ciliary length measurements were also done in primary rat hippocampal cells (Fig. 2B). (TIF) [file pone.0020580.s006.tif]

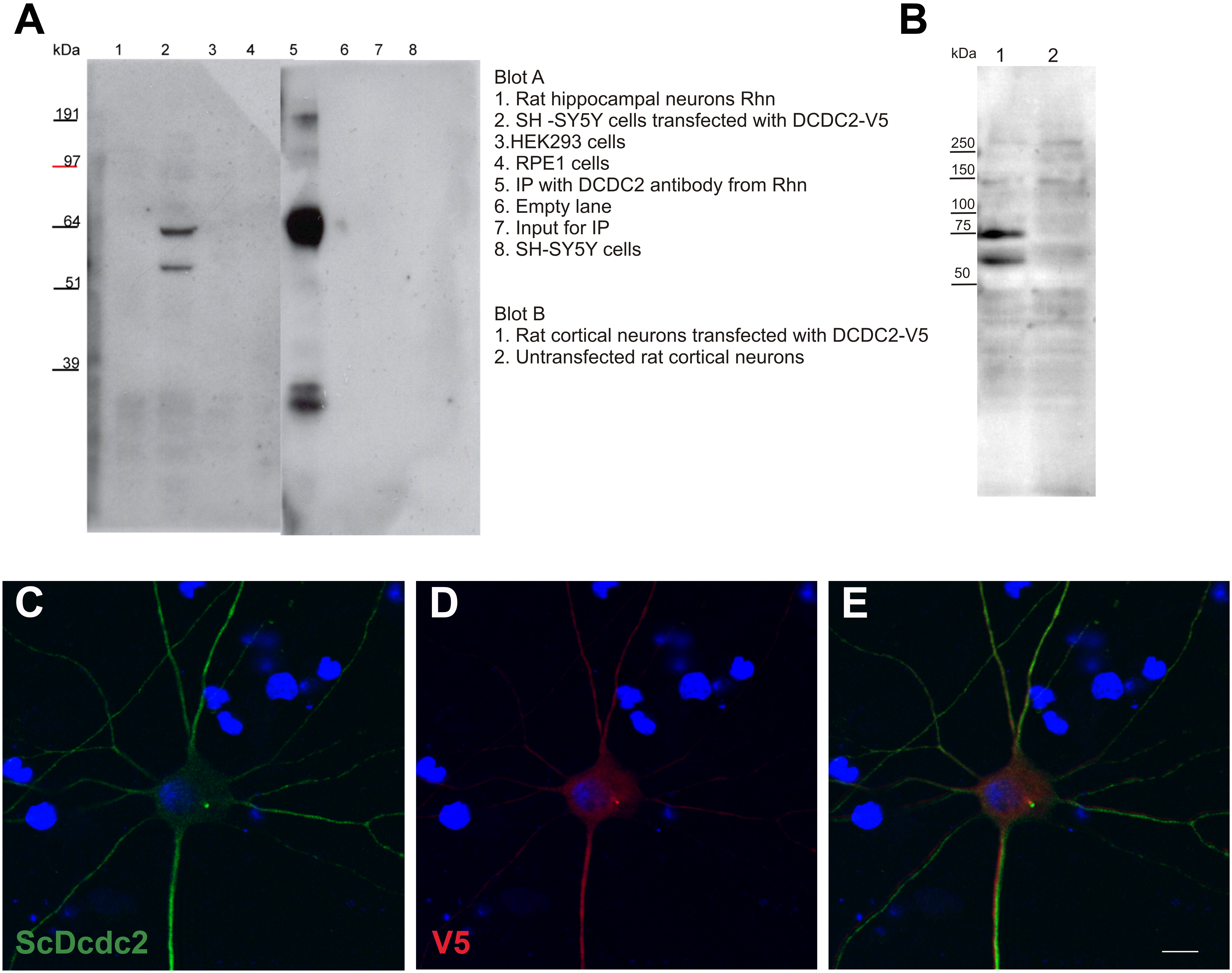

Supplement: Figure S6 — Verification of the specificity of the DCDC2 antibody (sc-50728 Santa Cruz Biotechnology). Detection of Dcdc2 after immunoprecipitation with DCDC2 antibody (blot A lane 5). Detection of DCDC2 in SHSY5-cells (blot A lane 2) and rat primary cortical neurons (blot B lane 1) after overexpression of DCDC2. Immunocytofluorescent staining showing that Dcdc2 antibody recognizes the overexpressed form of DCDC2-V5 by co-staining with the tag marker V5 (C–E). (TIF) [file pone.0020580.s007.tif]

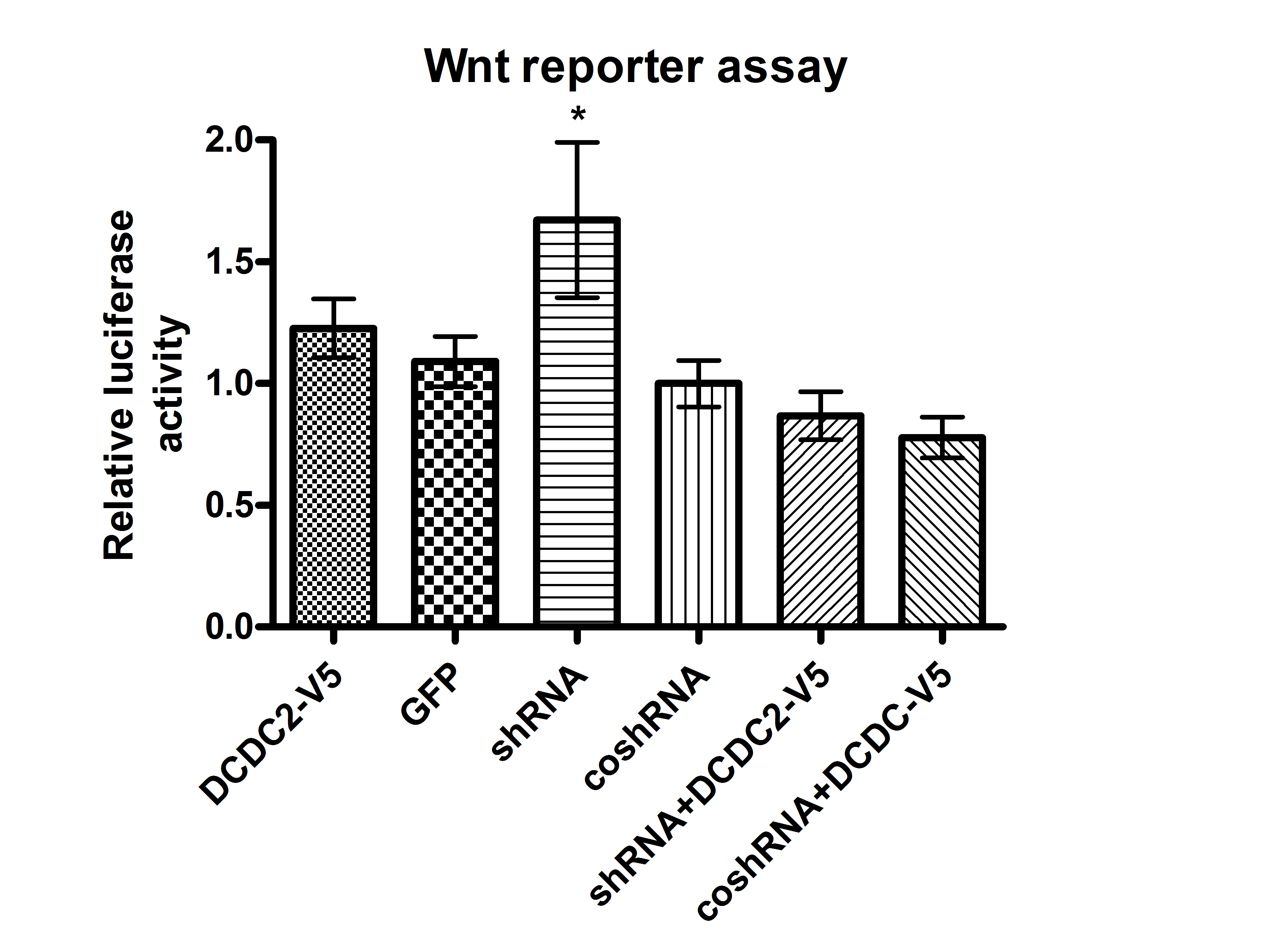

Supplement: Figure S7 — The increase in canonical Wnt signaling after Dcdc2 knock-down is inhibited by simultaneously expressing DCDC2-V5. Knocking-down Dcdc2 with shRNA leads to increased Wnt signaling as measured by a luciferase reporter assay, this increase can be blocked by simultaneously over-expressing DCDC2-V5 (* p<0,05, ANOVA, followed by T-test.) (TIF) [file pone.0020580.s008.tif]

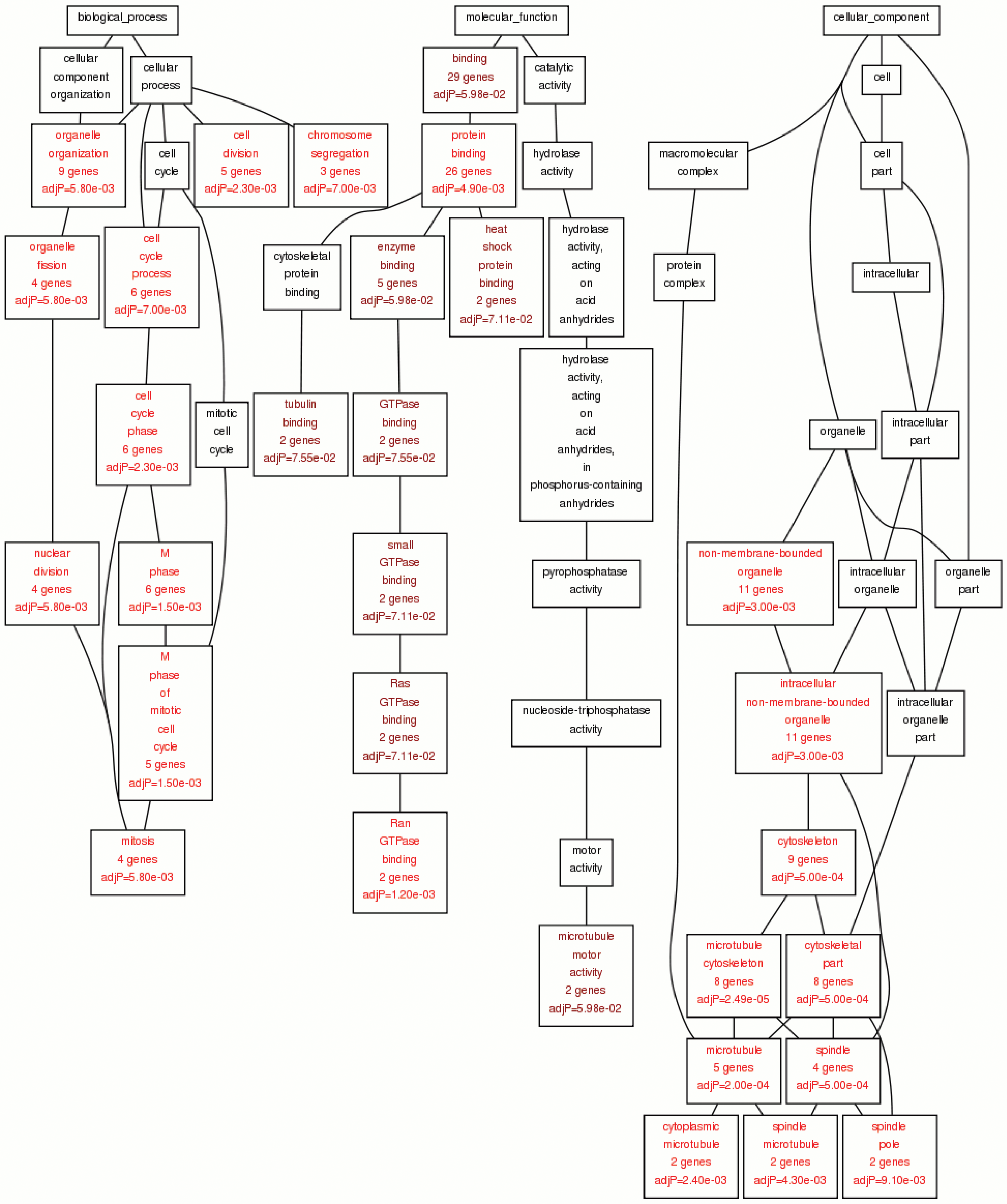

Supplement: Figure S8 — Gene ontology analysis of the microarray data of differentially expressed genes after DCDC2 overexpression. There is enrichment in GO terms of the cellular component in microtubule cytoskeleton (adjusted p-value 2.49×10^−5^) as well as in biological processes related to cell cycle. (TIF) [file pone.0020580.s009.tif]

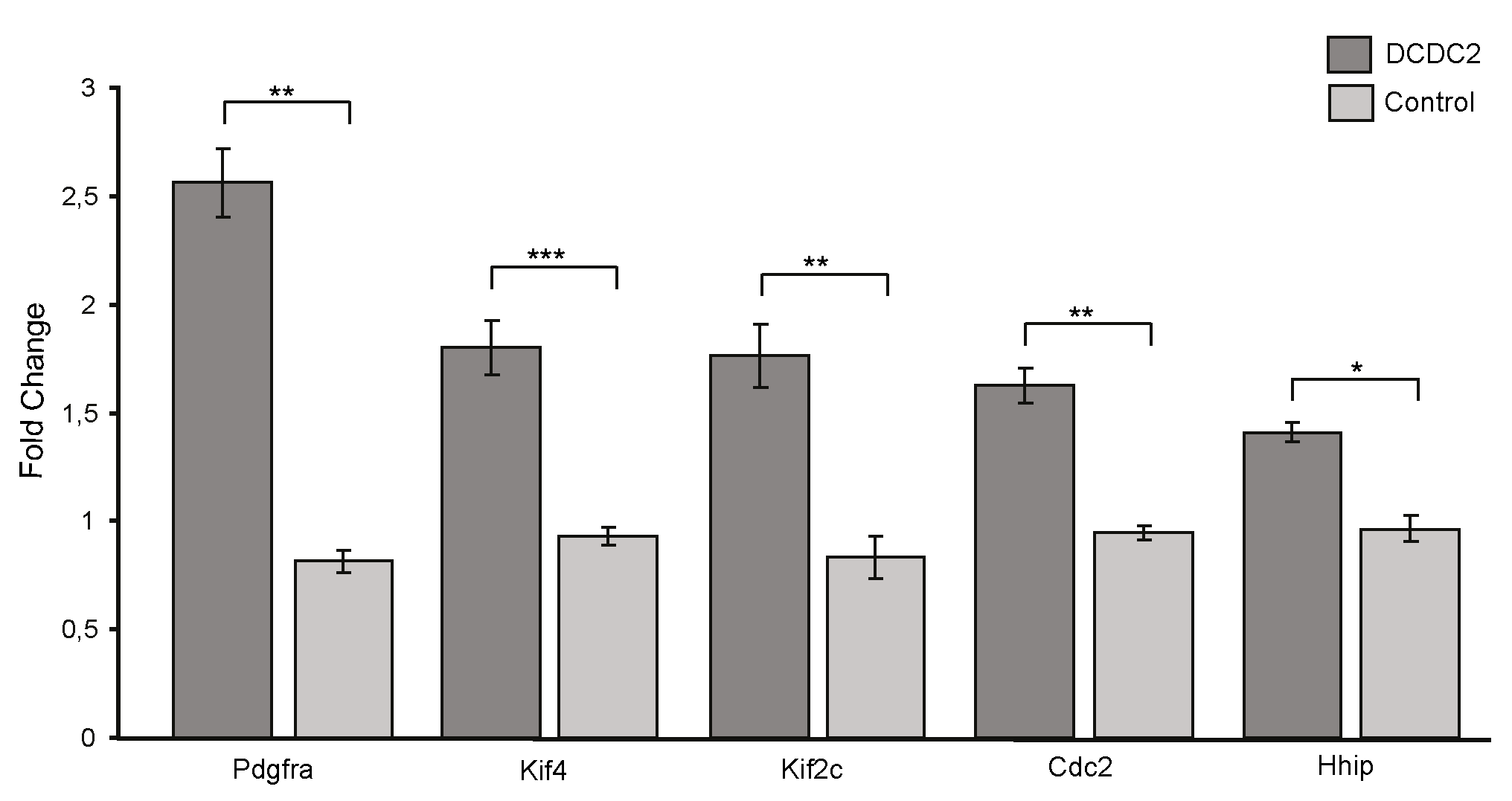

Supplement: Figure S9 — Differential expression of five genes upregulated upon DCDC2 overexpression in hippocampal neurons. The mRNA expression levels were measured by qRT-PCR in triplicates from eight technical replicates. Relative expression levels of the genes in the DCDC2 overexpressing neurons were compared to expression levels in control transfected neurons using Ppia as endogenous control and using ΔΔCt method (mean fold-change ± SEM). Similar results were obtained when Hprt was used as endogenous control. Significance was tested using Student's t-test using ΔCt values (***p<0.005, ** p<0.01 ja *p<0.05). (TIF) [file pone.0020580.s010.tif]

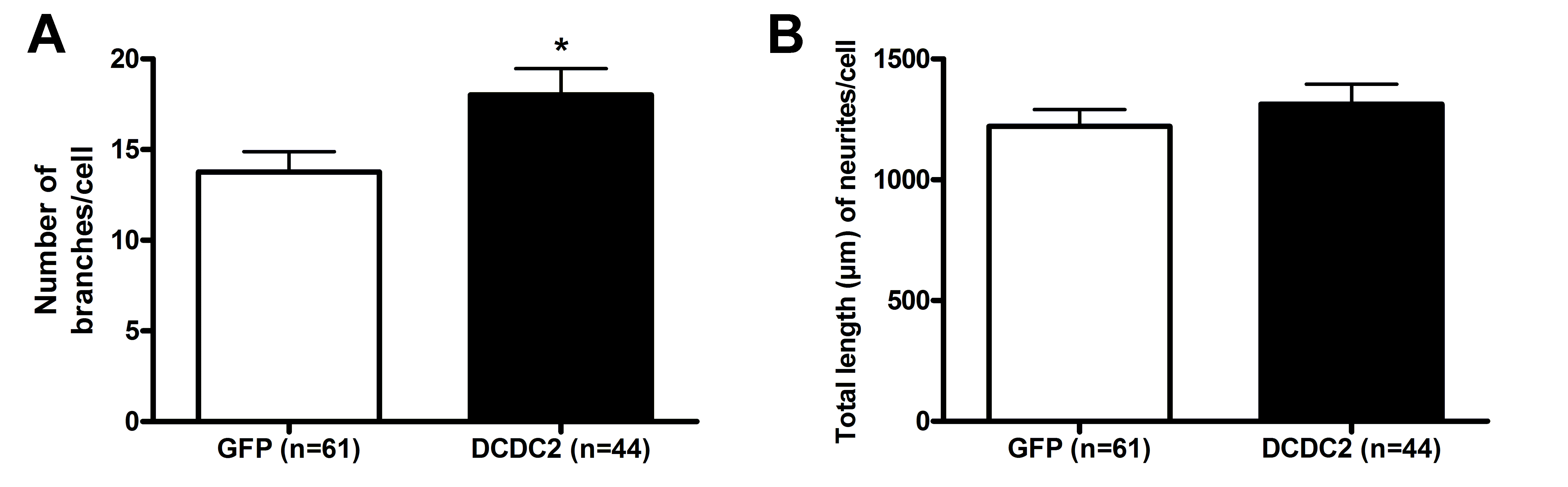

Supplement: Figure S10 — Overexpression of DCDC2 leads to increased branching in rat hippocampal neurons. Cells were transfected with either DCDC2-V5 and GFP or GFP only before plating. The morphology was analyzed by measuring length and branching of neurites using NeuronJ plug-in of ImageJ. Branching (A) but not total length (B) was significantly changed after overexpression of DCDC2 (*p<0,05, T-test using Welch correction, DCDC2-V5 n = 44, GFP n = 61). (TIF) [file pone.0020580.s011.tif]
